# Optimizing isothiocyanate formation during enzymatic glucosinolate breakdown by adjusting pH value, temperature and dilution in *Brassica* vegetables and *Arabidopsis thaliana*

**DOI:** 10.1038/srep40807

**Published:** 2017-01-17

**Authors:** Franziska S. Hanschen, Rebecca Klopsch, Teresa Oliviero, Monika Schreiner, Ruud Verkerk, Matthijs Dekker

**Affiliations:** 1 Department of Plant Quality, Leibniz Institute of Vegetable and Ornamental Crops, Theodor-Echtermeyer-Weg 1, 14979 Grossbeeren, Germany; 2 Food Quality and Design Group, Wageningen University, PO Box 17, 6700 AA Wageningen, The Netherlands

## Abstract

Consumption of glucosinolate-rich Brassicales vegetables is associated with a decreased risk of cancer with enzymatic hydrolysis of glucosinolates playing a key role. However, formation of health-promoting isothiocyanates is inhibited by the epithiospecifier protein in favour of nitriles and epithionitriles. Domestic processing conditions, such as changes in pH value, temperature or dilution, might also affect isothiocyanate formation. Therefore, the influences of these three factors were evaluated in accessions of *Brassica rapa, Brassica oleracea,* and *Arabidopsis thaliana*. Mathematical modelling was performed to determine optimal isothiocyanate formation conditions and to obtain knowledge on the kinetics of the reactions. At 22 °C and endogenous plant pH, nearly all investigated plants formed nitriles and epithionitriles instead of health-promoting isothiocyanates. Response surface models, however, clearly demonstrated that upon change in pH to domestic acidic (pH 4) or basic pH values (pH 8), isothiocyanate formation considerably increases. While temperature also affects this process, the pH value has the greatest impact. Further, a kinetic model showed that isothiocyanate formation strongly increases due to dilution. Finally, the results show that isothiocyanate intake can be strongly increased by optimizing the conditions of preparation of Brassicales vegetables.

Brassicales plants, including *Arabidopsis thaliana*, broccoli, cabbage and pak choi contain glucosinolates (GLSs). These sulphur-containing secondary plant metabolites not only play an important role in the plant’s defense against biotic stressors, but also affect the taste of these vegetables and contribute to human health^1^. To date, a total of 130 GLSs have been identified that can be subdivided according to the structure of their variable side chain into the group of aliphatic, aromatic or indole GLS^2^. These plant metabolites are stored in the plant vacuole or in specialized S-cells^3^, but upon cell disruption, they encounter the endogenous GLS-hydrolysing enzyme myrosinase, a *β*-d-thioglucosidase, and the GLSs will be hydrolysed to release isothiocyanates (ITCs), nitriles and epithionitriles (EPTs)^4^.

Due to their electrophilicity, GLSs in general and ITCs in particular act as effective deterrents against a multitude of pathogens as these compounds have antifungal, antimicrobial as well as insecticidal properties^5^,^6^,^7^. For humans, ITCs, such as the 4-(methylsulphinyl)butyl ITC (4MSOB-ITC), exert a multitude of health beneficial effects, including antimicrobial, antiinflammatory, antithrombotic and chemopreventive effects^8^,^9^,^10^. In additional, consumption of *Brassica* vegetables has been associated with a decreased risk to several types of cancer^10^,^11^,^12^. On the other hand, nitriles and EPTs were shown to have less health beneficial potential^5^,^13^,^14^ and even to exert some harmful effects, especially in animal nutrition^15^. Therefore, in respect to a healthy human diet, it is of great interest to provide food rich in ITCs with low levels of nitriles and EPTs.

To better understand the complexity of generating ITC-rich foods, it is of great importance to comprehend the enzymatic hydrolysis process of GLS. When myrosinase and GLS come into contact, d-glucose is cleaved and an instable aglucon, the thiohydroximate-*O*-sulphate, is released. l-Ascorbate is a cofactor for myrosinase since it promotes the rate-limiting step of the reaction, i.e. the release of the glucose molecule from the active site of myrosinase^16^. The aglucon is unstable and spontaneously rearranges either via a Lossen rearrangement to form the ITC, the pungent principle of, e.g. mustard, or degrades releasing sulphur to form a nitrile^17^,^18^. Some ITCs, such as indole-ITC or ITC from 2-hydroxyalkenyl GLS, are very instable and form the respective indole-3-carbinols by release of thiocyanate anion (SCN^−^) or cyclize to the respective oxazolidine-2-thiones^17^. The spontaneous formation of nitriles is favoured at low pH values, as protons block the Lossen rearrangement^18^. Further, if an alkenyl GLS and the epithiospecifier protein (ESP) are present, the degradation process of the aglucon can be modified and an EPT can be released^17^,^19^ (Fig. 1). Moreover, the ESP has also been identified to favour nitrile formation from other (non-alkenyl) GLS^17^,^20^. The activity of this protein was shown to be dependent on ferrous ions (Fe^2+^)^21^. In additional, in *A. thaliana*, other modifying proteins can be present and the so-called nitrile specifier proteins (NSPs) also play a role in the degradation of the aglucon. Thus, in the presence of NSPs, the formation of nitriles is favoured, whereas the epithiospecifier modifier protein (ESM) blocks EPT and nitrile formation to promote ITC formation^22^. On the other hand, a fourth class of specifier proteins, namely the thiocyanate-forming protein (TFP), was reported to catalyse the formation of benzylthiocyanate in *Lepidium sativum*^25^. As many *Brassica* vegetables, among them broccoli, cabbage and pak choi, contain ESP, nitriles and EPTs instead of ITCs can be the resultant main breakdown products^14^,^26^,^27^. Moreover, many *Brassica* vegetables are thermally processed prior to consumption and this changes ITC formation dramatically. Whereas mild heat treatment was shown to inhibit the activity of the thermally labile ESP and thus favours ITC formation^28^, more intense heat treatment also inactivates the myrosinase^29^,^30^. However, then GLS can be still degraded because of thermal degradation, but mainly nitriles will be formed^31^,^32^,^33^, while the ITCs will be degraded due to the heat^34^. Moreover, during cooking, GLS content of vegetables often decreases due to leaching effects^35^. Thus, thermal processing often reduces ITC formation and uptake^36^,^37^. Ingested intact GLS, however, can also be degraded by human microbial myrosinases in the colon, and subsequently, ITC can be formed^38^.

Therefore, enzymatic hydrolysis of GLS plays an important role in the biofunctionality of GLS breakdown product related cancer prevention. However, additional factors, e.g. the pH value during vegetable slicing or also in the mouth during chewing, as well as the preparation temperature of the vegetable, might also affect the enzymatic hydrolysis and subsequent reaction pathways, and ultimately, the ITC concentration. Therefore, the aim of the present study was to evaluate the effect of (1) “refrigerator” (4 °C), ambient (22 °C) and “chewing” temperature (37 °C) as well as (2) acidic (pH 4), medium (pH 6) and slightly basic pH values (pH 8), that are relevant for food preparation, on the profile of enzymatic breakdown products of GLS in selected *Brassica* vegetables. For this purpose, *Brassica rapa* and *Brassica oleracea* were compared to the model plant *A. thaliana*. The two *B. rapa* accessions (B. *rapa* ssp. *rapa* and *B. rapa* ssp. *oleifera*) were chosen because of their different content in unsaturated aliphatic GLS (pre-experiment), whereas BroccoCress® (*B. oleracea* var. *italica*) was selected due to its GLS profile and popularity as a mini green vegetable. In addition, selected accessions of the model plant *A. thaliana* (Bur-0, Hi-0) were chosen as this plant is well characterized for its GLS hydrolysis proteins (e.g. ESP, ESM, NSP 1–5^39^). Bur-0 and Hi-0 are both rich in 2Prop-GLS, but differ in their hydrolysis behaviour. In detail, Hi-0 forms ITCs, while Bur-0 releases EPTs^40^. Moreover, we also evaluated whether the hydrolysis process of GLS in *Brassica* vegetables can be modified by dilution with water, artificial saliva or heat-treated plant homogenate, e.g. by changing the concentration of GLS, myrosinase and specifier proteins present in the environment during GLS hydrolysis. Next to the *B. rapa* plants and the BroccoCress® mini greens of white cabbage (*B. oleracea* convar. *capitata* var. *alba*) were also evaluated since they differ in their GLS profile compared to BroccoCress®. The results of these studies were modelled, in order (1) to determine the impact of pH, temperature and dilution and to compare the various species belonging to the Brassicaceae family as regards their GLS metabolism as well as (2) to obtain knowledge on the kinetics of the hydrolysis and breakdown reactions as a first step in optimizing ITC formation, and thus intake, during the food preparation process.

## Results

In addition to quantification of the GLS breakdown products under the different pH, temperature and dilution conditions, the plant materials were evaluated for their GLS content, myrosinase activity, ESP activity and l-ascorbic acid (vitamin C) content.

### Glucosinolates in *Brassica rapa, Brassica oleracea* and *Arabidopsis thaliana*

In the six different plants, 16 different GLSs were detected with aliphatic GLSs being the most abundant (see Supplementary Information). *B. rapa* BR215 and BR374 mainly contained 3-butenyl GLS (3But-GLS) (0.3 and 1.1 μmol/g fresh weight (FW)) and 4-pentenyl GLS (0.2 and 0.4 μmol/g FW), whereas the *B. oleracea* BroccoCress® was rich in 4-(methylsulphinyl)butyl GLS (4MSOB-GLS) and 4-(methylthio)butyl GLS (4MTB-GLS) (5.9 and 1.1 μmol/g FW, respectively). The white cabbage sprouts mainly contained 2-propenyl GSL (2Prop-GLS) and 3-(methylsulphinyl)propyl GLS (3MSOP-GLS) (1.2 and 0.6 μmol/g FW, respectively). The *A. thaliana* plants were rich in alkenyl GLS: Bur-0 had high levels of 3But-GLS and 2Prop-GLS (2.9 and 1.8 μmol/g DW), while Hi-0 was only rich in 2Prop-GLS (5.1 μmol/g DW). With regard to the GLS content in the heat-treated plant homogenates (inactivated plant material), the same main GLSs were detected as compared to the non-treated material. While myrosinase-inactivated BR215 and BR374 had lower GLS concentrations compared to the native material, myrosinase-inactivated BroccoCress® had the same GLS concentrations and white cabbage sprout material had even higher extractable concentrations.

### Myrosinase activity, ESP activity and l-ascorbic acid content

Myrosinase activity, ESP activity as well as l-ascorbic acid content differed between the plants (Table 1). Bur-0 followed by BroccoCress® had the highest myrosinase activities (MYR), whereas white cabbage sprouts had the lowest myrosinase activity. With respect to ESP activity, *A. thaliana* Bur-0 also had the highest percentage of 1-cyano-2,3-epithiopropane (CETP) relative to all degradation products from 2Prop-GLS followed by BroccoCress®, while the *A. thaliana* Hi-0 had the lowest ESP activity. The *B. rapa* accession BR215 and BR374 plants as well as white cabbage sprouts had medium ESP activities. With regard to the ratio of ESP/myrosinase activity (ESP/MYR), white cabbage sprouts had the highest ratio, followed by BR215, BR374, Bur-0 and BroccoCress® plants which had a ratio of 7 to 17-times less. For Hi-0, the ratio was more than a 100-fold less compared to the white cabbage sprouts. With regard to the overall activity of myrosinase per μmol of (total) GLS (MYR/GLS), again Bur-0 had the highest (79 U/μmol), followed by BR215, BroccoCress®, BR374 and Hi-0 (50, 43, 17 and 15 U/μmol, respectively). In contrast, white cabbage sprouts had a distinct lower amount of myrosinase per μmol of GLS (0.9 U/μmol). With respect to ESP activity relative to the GLS content (ESP/GLS), again, Bur-0, together with BR215, had the highest level, followed by BroccoCress®, BR374 and the white cabbage sprouts, while the ESP/GLS ratio of Hi-0 was lowest. All Brassicaceae plants used for the experiment on the effect of pH and temperature were also analysed for their l-ascorbic acid content as this is an important cofactor for myrosinase. The l-ascorbic acid concentration ranged from 0.6 to 31.5 mg/100 g FW. While BR215 had the highest l-ascorbic acid content, white cabbage sprouts had the lowest (Table 1).

### Glucosinolate breakdown products in *B. rapa, B. oleracea* and *A. thaliana*

Upon cell disruption, GLS in *Brassica* and *Arabidopsis* plants are hydrolysed by myrosinase and resulting ITCs, nitriles or EPTs are formed. In the six plants analysed, a total of 26 breakdown products were identified (see Supplementary Information). Upon hydrolysis (at 22 °C with a water/plant ratio of 2:1), all plants except for Hi-0, formed mainly EPT and nitriles, whereas Hi-0 mainly produced ITC. Nevertheless, BroccoCress® had the highest concentration of total ITC, which was mainly attributed to the cancer preventive compound, 4MSOB-ITC (sulforaphane). In general, the two *B. rapa* accessions BR215 and BR274 and Bur-0 mainly formed the EPT 1-cyano-3,4-epithiobutane (CETB), with BR374 and Bur-0 being very rich in this GLS breakdown product (more than 1 μmol/g FW). While BR374 also contained noteworthy amounts of the corresponding 3-butenyl ITC (3But-ITC), Bur-0 was also rich in CETP, that was also the main breakdown product found in white cabbage sprouts. In contrast, Hi-0 mainly formed the corresponding 2-propenyl isothiocyanate (2Prop-ITC). Finally, BroccoCress® was a producer of nitriles and rich in 5-(methylsulphinyl)pentanenitrile (4MSOB-CN) and 5-(methylthio)pentanenitrile (4MTB-CN). Nevertheless, it also contained 0.6 μmol of 4MSOB-ITC.

### Effect of temperature and pH value on the enzymatic hydrolysis of glucosinolates

In many *Brassica* vegetables, most GLSs form mainly EPT and nitriles instead of ITC^14^,^27^. It is thus of great interest to identify factors that might shift the breakdown towards ITC formation.

During food processing, the temperature of the vegetables or a modified pH value might have an effect on which GLS breakdown products are formed. Therefore, by adding a solution of 0.1 M acetic acid (acidic) or 0.1 M sodium carbonate (alkaline), the pH value was modified and effects were tested in combination with the typical domestic preparation temperatures of 4, 22 and 37 °C.

With regard to the endogenous plant’s pH values, BroccoCress® had the lowest pH value (pH 5.6), while BR215, BR374 and Bur-0 had a pH value of 6.3, and Hi-0 of 6.4. After addition of the acidic or alkaline solution, the pH values dropped or increased. Thus, the corresponding acidic pH value of the plants was 3.6 (BroccoCress®) or 4.2 for the other Brassicaceae plants, whereas the corresponding basic pH value was 8.0 (Bur-0), 8.2 (Hi-0, BroccoCress®) or 8.3 (BR215 and BR274).

#### Response surface modelling

To determine the effect of the independent parameters of pH value and temperature on the main GLS breakdown products, response surface modelling was performed using the relative amounts (%) of the ITC, nitrile and EPT derived from the main GLS. The obtained data for the models are presented in Table 2. For all main compounds of the plants, significant response surface models could be calculated, except for the ITC and EPT deriving from the main GLS of Bur-0 and for 4MTB-GLS hydrolysis in BroccoCress®, (Table 2). As a basic equation for all response surface models, the following equation 1 can be given:


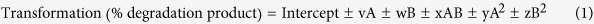


with A being the pH and B being the temperature.

With two exceptions, quadratic or reduced quadratic (without one or two of the coefficients) models were calculated for the breakdown products of the main GLS in these plants: For Hi-0-derived CETP, a 2Fl-model (2-factor interaction; without quadratic terms) was used, while for 4-pentenylnitrile (3But-CN) in Bur-0, a linear model was employed (Table 2) (without quadratic terms, without interaction of A and B).

With regard to the *B. rapa* accessions, namely BR215 and BR374, the 3D models obtained for 3But-ITC, 3But-CN and CETB are similar, and thus, only the 3D model graphs for BR215 are shown in Fig. 2. The results for 2Prop breakdown products for Hi-0 are presented in Fig. 3 and the effect of pH and temperature on 4MSOB-GLS degradation in BroccoCress® is depicted in Fig. 4.

In all plants except Bur-0, the pH value had a great impact on ITC formation. While at the endogenous plant’s pH value, the formation of EPTs (BR215, BR374) (Fig. 2C) or nitriles (BroccoCress®) (Fig. 4B) was very prominent, at acidic or alkaline conditions, the formation of ITCs increased (compare Figs 2A, 3A and 4A). In Hi-0, although mainly 2Prop-ITC was formed, low and high pH increased the percentage of 2Prop-ITC (Fig. 3A) and reduced 3-butenenitrile (2Prop-CN, Fig. 3B). The formation of nitriles in the presence of high EPT concentrations was differently affected in the plants. While in BR215, an acidic pH increased nitriles considerably (Fig. 2B), the effect was less pronounced on the main nitriles of BR374 or Bur-0 (Table 2). Similar to the pH value, temperature also significantly affected the formation of ITCs, EPTs and nitriles and interactive effects of pH and temperature were also present (Table 2). In BR215, BR374, Hi-0 and BroccoCress®, formation of ITC and EPT was affected by the temperature, but the effect was distinctly lower compared to the effect of pH (compare Figs 2, 3 and 4 and Table 2). For example, models identified that in BR215 at both acidic and alkaline pH, 3But-ITC formation is highest at lower ambient temperature (16–18 °C) and decreases at high or low temperatures (Fig. 2A). In contrast, in BroccoCress® at a given pH value, 4MSOB-ITC increases with higher temperature (Fig. 4A). In Hi-0, the effect of temperature on ITC and nitrile formation is limited, but has an effect on CETP formation, which is increased at acidic pH with rising temperature (Fig. 3C). Although there was no suitable model for Bur-0, at acidic pH, the percentage of the nitriles and ITC from 2Prop-GLS and 3But-GSL at 37 °C were higher compared to the other temperatures, while the percentage of EPT was lower (see Supplementary Information, tested by one-way ANOVA and Tukey’s HSD test, STATISTICA version10). With respect to optimized ITC formation, the models predicted optimal ITC formation in BroccoCress® and Hi-0 to be at basic pH value and 37 °C (Figs 3A and 4A), while in BR215 acidic pH and 4 °C are the optimal conditions for ITC formation (Fig. 2A). In BR374, ITC formation could be optimized both at acidic and alkaline pH value at lower ambient temperature (16–18 °C).

### Effect of dilution on the enzymatic hydrolysis of glucosinolates

To identify additional optimization strategies for ITC formation during the enzymatic hydrolysis of GLS, we tested whether different dilution formulas and intensities have an influence on the type of GLS breakdown products. Note that at lower ESP concentration, the spontaneous, non-catalysed formation of ITC from the aglucon might be favoured because the probability should decline that the ESP meets the aglucon. Thus, we studied the following dilution patterns: (1) fresh plant material was diluted with water at 22 °C as relevant for juice preparation; (2) artificial saliva was used at 37 °C, thereby simulating hydrolysis under chewing conditions; and (3) dilution with myrosinase- and ESP-inactivated plant material at 22 °C, thus imitating hydrolysis in cooked samples, e.g. when cooked and fresh materials are mixed during food preparation. The inactivated plant material still contained GLS (see Supplementary Information), while breakdown products were usually present only in very small amounts. The concentrations of breakdown products in the myrosinase inactivated materials are presented in the Supplementary Information. In the inactivated material of the two *B. rapa* only 3But-ITC was present in low amounts (0.09% and 3.1% compared to the 3But-GSL hydrolysis products of fresh tissue). In the inactivated white cabbage sprouts traces of 2Prop-CN (0.8% compared to the 2Prop-GSL hydrolysis products in fresh tissue) and of 2Prop-ITC (0.08% compared to the 2Prop-GSL hydrolysis products in fresh tissue) were present. In myrosinase inactivated BroccoCress®, in comparison slightly higher amounts of nitriles and traces of ITC were present: 4MTB-CN (6.8% compared to the 4MTB-GSL hydrolysis products of fresh tissue), 4MTB-ITC (0.42% compared to the 4MTB-GSL hydrolysis products of fresh tissue) and 4MSOB-CN (9.1% compared to the 4MSOB-GSL hydrolysis products of fresh tissue) were detected. Thus, here in the BroccoCress®, approx. up to 8% of the 4MSOB-CN formed in the highest dilution level could be attributed to the basis level originated from the inactive material. Upon hydrolysis of the GLSs for all three diluents types, mainly EPTs and nitriles were formed at low dilution with the notable exception of the experiment using saliva and BroccoCress®. The determined absolute concentrations of hydrolysis products are presented in the Supplementary Information. For all diluents and in all plants studied, absolute EPT or nitrile concentrations decreased with increasing dilution and (except in BR215 where no effect on absolute ITC concentrations was observable) ITC concentrations increased (compare Supplementary Information). To generate models to predict the effect of various dilution patterns as well as to obtain additional information on the kinetics of the reactions involved in GLS hydrolysis, modelling was performed using the percentages of the respective breakdown products formed from the corresponding main GLS.

**Figure 1:**
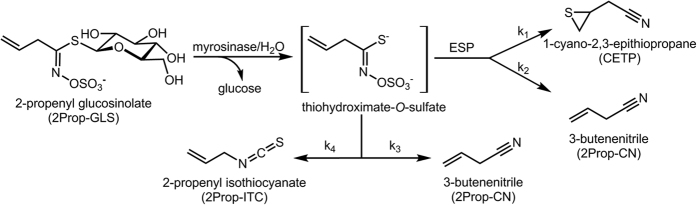
Enzymatic hydrolysis pathways of 2-propenyl glucosinolate (2Prop-GLS) to 2-propenyl isothiocyanate (2Prop-ITC), 3-butenenitrile (2Prop-CN) and 1-cyano-2,3-epithiopropane (CETP). K_1_–k_4_ represent the ratios of the rate constants of the reactions. ESP: epithiospecifier protein.

On the basis of the reactions presented in Fig. 1, models for breakdown product formation from EPT-forming alkenyl-GLS were evolved. The reaction constants k_1–4_ were labelled according to the degradation reactions presented in Fig. 1. The following equations were used for the *B. rapa* accessions BR215 and BR374, and white cabbage sprouts. The kinetic Excel-model pointed out that k_3_ = 0. Therefore, equations could be simplified and either k_1_ and k_4_ or x_1_ = k_1_/k_2_ and x_4_ = k_4_/k_2_ could be calculated.


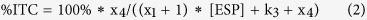



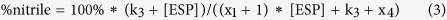



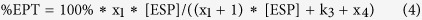



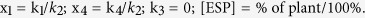


For the non-EPT-forming BroccoCress®, the model equations for 4MSOB-GLS and 4MTB-GLS breakdown could be simplified:


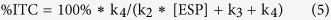



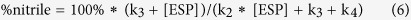



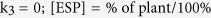


The results are presented in Table 3. In Fig. 5, the models obtained for the effect of dilution on the hydrolysis of 2Prop-GLS in white cabbage sprouts and 4MSOB-GLS in BroccoCress® are presented.

Upon dilution, ITC formation increased in all experiments. This increase was inversely correlated with the corresponding EPT formation (Fig. 5A–C) or for BroccoCress®, with the formation of the respective 4MSOB-CN (Fig. 5D–F) and 4MTB-CN. For example, upon dilution with water (17-fold dilution compared to 2-fold), 3But-ITC content in BR215 and BR374, increased by 8.9- and 3.1-fold, respectively. In BroccoCress®, 4MSOB-ITC increased by 2.4 fold and in white cabbage sprouts 2Prop-ITC increased by 2.1-fold (see Supplementary Information). With regard to the three diluent types, ITC formation, even at the lowest dilution level (2-fold dilution), differed. In detail, ITC concentration was highest in the saliva buffer (4MSOB-ITC in BroccoCress®, Fig. 5E) or in the myrosinase-inactivated plant material (BR215, see Supplementary Information; white cabbage sprouts, Fig. 5C; 4MTB-ITC in BroccoCress®, see Supplementary Information), while the ITC concentration in the diluent water was usually lowest (see Supplementary Information, Fig. 5A,D).

Moreover, the degradation of GLS was differently affected by the diluents and in white cabbage sprouts, the highest 2Prop-ITC concentrations were formed upon hydrolysis and dilution with myrosinase-inactivated white cabbage material (Fig. 5C). The models obtained for the effect of dilution on ITC formation in BroccoCress® were similar to the white cabbage sprouts (compare Fig. 5A–C with 5D–F), with the exception of artificial saliva for which 4MSOB-ITC was the main breakdown product of 4MSOB GLS (Fig. 5E). The hydrolysis of 4MTB-GLS in BroccoCress® to ITC was similarly affected by the diluents compared to 2Prop-GLS hydrolysis in white cabbage sprouts (Table 3). The models for the *B. rapa* accessions BR215 and BR374 were highly correlated and therefore less significant (Table 3).

With regard to the kinetics of the reaction rate, constants k_1_ and k_4_ (model BroccoCress®) and x_1_ = k_1_/k_2_ and x_4_ = k_4_/k_2_ (other dilution plant models) could be calculated. In all models, x_1_ or k_2_, both indicating the ESP-dependent reaction to the respective EPT or nitrile, were higher compared to x_4_ or the respective k_4_-value (indicating ITC formation) (Table 3). Between the diluents, significant differences between the rate constants were observed for BroccoCress® and for white cabbage sprouts. The factor x_4_ or the respective k_4_ constant of the diluent water were always lower than those of the other diluents. In white cabbage sprouts, x_1_ was two times larger in the water compared to the other diluents and the x_4_ value was 3.9 times higher in the saliva buffer and 5.5 times greater in the myrosinase-inactivated plant material compared to the diluent water. In BroccoCress®, k_2_ constants of 4MSOB-GLS and 4MTB-GLS (ESP-mediated reaction to the nitrile) were equal among the diluents (around 1), whereas the k_4_ constant of 4MSOB-GLS was 12.4-fold higher and 4MTB-GLS was only 4-times increased in the saliva buffer compared to the k_4_ constant of the water. When diluted with myrosinase-inactivated BroccoCress® material, k_4_ of 4MSOB-GLS was 2.6-fold higher and k_4_ of 4MTB-GLS was 6.8-fold higher compared to k_4_ of the diluent water (Table 3).

## Discussion

Consumption of *Brassica* vegetables contributes to a healthy diet due partly to their high GLS content. However, the cancer-preventing effects from these vegetables are strongly associated to their ability to release ITC^36^,^41^. As many *Brassica* vegetables comprise ESP that ultimately leads to EPT and nitrile formation^21^, ESP activity, and thus, EPT and nitrile formation changes upon plant development^42^,^43^. Moreover, ESP transcripts, and thus levels, can be induced by herbivore feeding^19^. While generalist insects usually prefer nitrile-forming plants, specialist insects often are attracted by ITC^17^,^44^. Therefore, modifier proteins like ESP probably enable plants to protect themselves from specialized insects to some extent.

As EPT and nitriles are less health promoting than ITCs and might even have adverse health effects^13^,^14^,^45^, it is of high interest to identify optimization strategies for the enzymatic breakdown of GLS towards ITC formation during food preparation. Therefore, in the present study, we evaluated how pH value, temperature and dilution can affect the enzymatic breakdown of GLS using selected *B. rapa* accessions, two varieties of *B. oleracea* micro greens, and as a model plant the well characterized *A. thaliana* accessions Bur-0 and Hi-0. As expected, the GLS profiles obtained in the present study matched with those reported in previous ones. In detail, BR215 and BR374 were rich in the aliphatic 3But-GLS and 4Pent-GLS, while being poor in indole and aromatic GLS^46^, and the GLS profiles in Bur-0 and Hi-0 matched the results reported by Witzel *et al*.^40^. In the present study, broccoli sprouts BroccoCress® contained mainly 4MSOB-GLS which is in line with other studies^47^,^48^. Finally, the white cabbage sprouts used in our study were rich in 2Prop-GLS – a finding that is consistent with Bellostas *et al*.^49^.

Upon enzymatic hydrolysis, the GLS in the different plant species released the corresponding breakdown products. With the exception of Hi-0, mainly EPTs were formed, or, as there were no alkenyl GLS, in BroccoCress®, nitriles were the main breakdown products. The observed formation of mainly EPTs and nitriles, or ITC, as was the case for Hi-0, is in line with previous studies on GLS breakdown in *A. thaliana* Hi-0 and Bur-0^40^, *B. rapa*^14^, white cabbage^50^ as well as broccoli^28^. This formation of high amounts of EPTs and nitriles at the expense of ITCs is based on the plant’s own ESP, which degrades the alkenyl-aglucons to EPT or other GLS-aglucons to nitriles^17^,^19^. The ESP is probably not a cofactor of myrosinase, but rather an enzyme catalysing the breakdown of the aglucon. This was demonstrated by Burow *et al*.^51^ in an experiment investigating the effect of vitamin C on ESP-mediated hydrolysis in *A. thaliana*^51^. While EPT formation, and thus ESP activity, was not affected due to the presence of l-ascorbic acid, ITC formation increased with increasing l-ascorbic acid, and thus, increasing myrosinase activity. These observations further support the growing evidence of a catalytic role for ESP^51^. However, without myrosinase to degrade the GLS to the aglucon, no GLS degradation will occur in presence of ESP^52^.

Regarding the mechanism of EPT formation, Brocker and Benn (1983) suggested an intramolecular mechanism for sulphur rearrangement^53^. Later, Foo *et al*.^54^ proposed a radical mechanism involving Fe^2+^ as a cofactor for ESP. In this mechanism, Fe^2+^ was proposed to transfer the sulphur of the *β*-d-thioglucosidic link to the alkenylic double bond in a non-stereo-selective manner, while simultaneously the cyano group is formed^54^. However, as radical scavengers and oxygen exclusion had no effect on EPT formation^51^, the mechanism for EPT formation still needs further investigation. In 2014, Brandt *et al*. developed molecular models in which the authors proposed the active sites and docking rearrangements of the aglucons for AtESP and two TFPs^55^. For all three specifier proteins, the model identified complexation of Fe^2+^ by the side chains of Asp, Glu and His, while the sulphate group is recognized by a conserved Arg and additional amino acid residues^55^.

**Figure 2:**
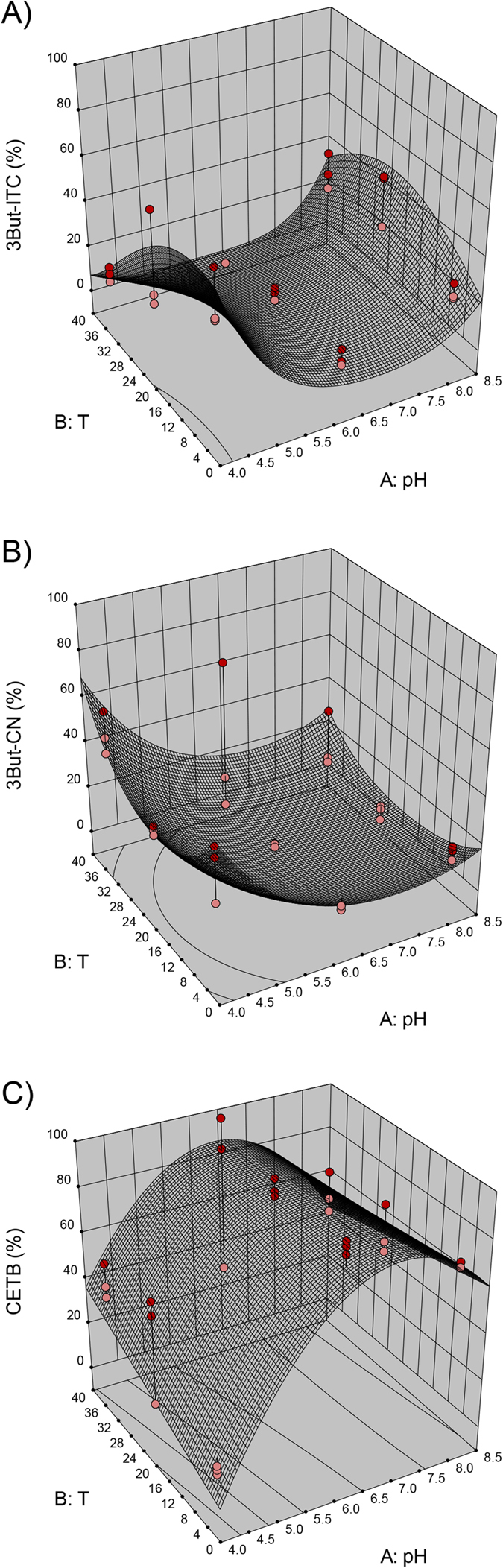
Quadratic and reduced quadratic response surface models for 3-butenyl glucosinolate (3But-GLS) hydrolysis to (**A**) 3-butenyl isothiocyanate (3But-ITC; quadratic model), (**B**) 4-pentenenitrile (3But-CN; reduced quadratic model) and (**C**) 1-cyano-3,4-epithiobutane (CETB; reduced quadratic model) in *Brassica rapa* BR215. Displayed are relative values (%) formed from the respective breakdown product relative to the corresponding 3But-GLS. Red dots indicate design above predicted model and pink dots indicate those values that are below the predicted model.

**Figure 3:**
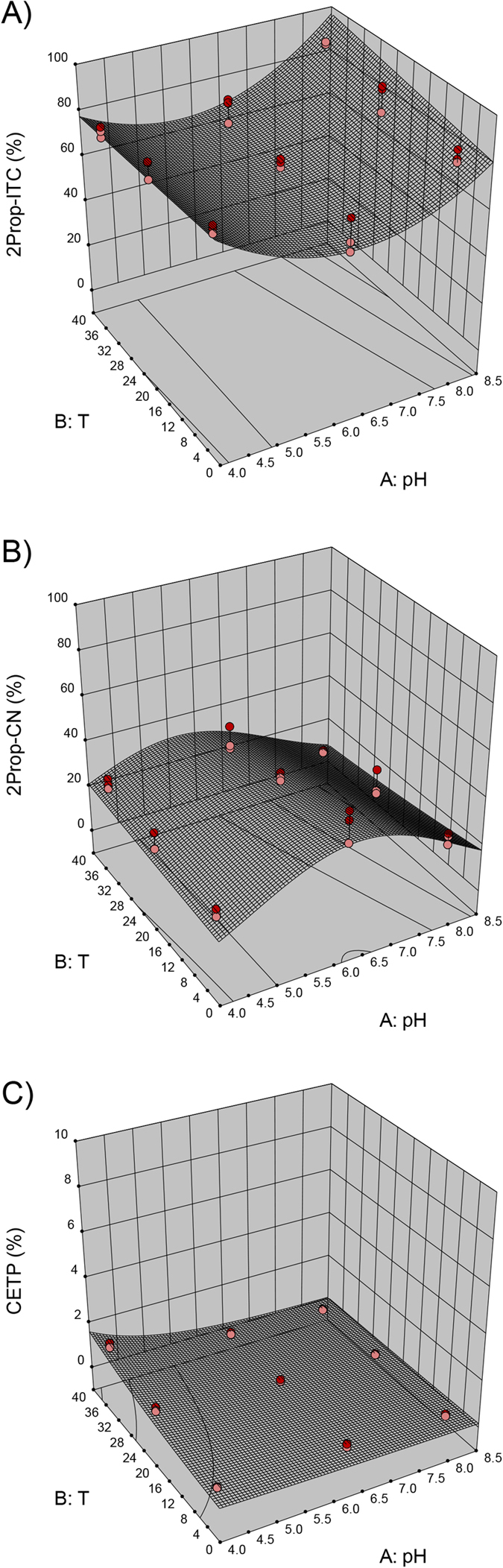
Reduced quadratic and 2-factor interaction (2FI) response surface models for 2-propenyl glucosinolate (2Prop-GLS) hydrolysis to (**A**) 2-propenyl isothiocyanate (2Prop-ITC; reduced quadratic model), (**B**) 3-butenenitrile (2Prop-CN; reduced quadratic model) and (**C**) 1-cyano-2,3-epithiopropane (CETP; 2FI model) in *Arabidopsis thaliana* Hi-0. Displayed are relative values (%) formed from the respective breakdown product relative to the corresponding 2Prop-GLS. Red dots indicate design above predicted model and pink dots indicate those values that are below the predicted model.

**Figure 4:**
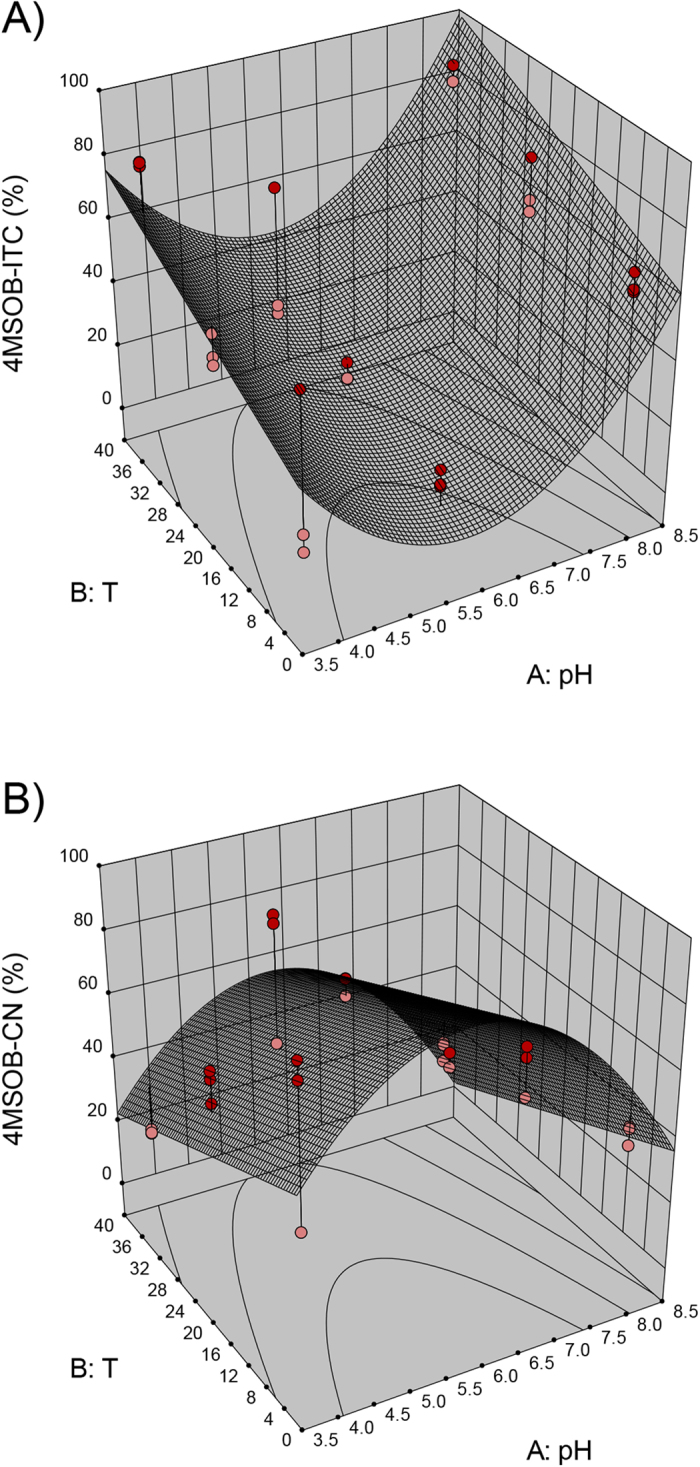
Reduced quadratic response surface models for 4-(methylsulphinyl)butyl glucosinolate (4MSOB-GLS) hydrolysis to (**A**) 4-(methylsulphinyl)butyl isothiocyanate (4MSOB-ITC; reduced quadratic model) and (**B**) 5-(methylsulphinyl)pentanenitrile (4MSOB-CN; reduced quadratic model) in *Brassica oleracea* BroccoCress®. Displayed are relative values (%) formed from the respective breakdown product relative to the corresponding 4MSOB-GLS. Red dots indicate design above predicted model and pink dots indicate those values that are below the predicted model.

**Figure 5:**
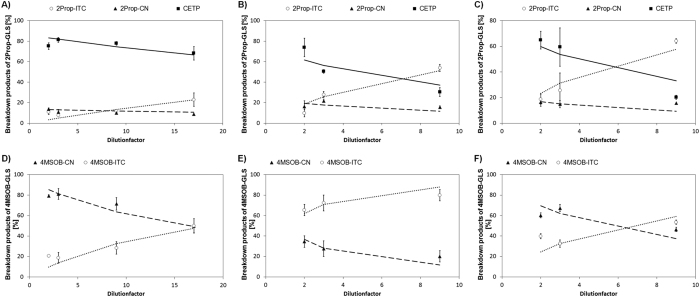
Kinetic models of the effect of dilution with (**A**) water at 22 °C, (**B**) artificial salivary fluid at 37 °C and (**C**) myrosinase-inactivated plant homogenate at 22 °C on 2-propenyl glucosinolate hydrolysis in white cabbage sprouts and of the effect of dilution with (**D**) water at 22 °C, (**E**) artificial salivary fluid at 37 °C and (**F**) myrosinase-inactivated plant homogenate at 22 °C on 4-(methylsulphinyl)butyl glucosinolate (4MSOB-GLS) hydrolysis in *Brassica oleracea* BroccoCress®. CETP: 1-cyano-2,3-epithiopropane; 4MSOB-CN: 5-(methylsulphinyl)pentanenitrile; 4MSOB-ITC: 4-(methylsulphinyl)butyl isothiocyanate; 2Prop-CN: 3-butenenitrile; 2Prop-ITC: 2-propenyl isothiocyanate. Black lines indicate the model for the epithionitrile, dashed lines represent the nitrile-model and dotted lines show the model for the respective isothiocyanate.

As EPTs and nitriles are found at high levels in many *Brassica* vegetables, it was of interest to identify strategies to influence the breakdown of GLS into health-promoting ITCs. By studying the effects of different pH values and temperatures, the optimal conditions were identified using response surface modelling approaches (Figs 2, 3 and 4). Although there were slight species-specific differences, the pH value affected ITC formation more than the temperature in all plants, except for Bur-0. At acidic (BR215, BR374) and alkaline pH values (BR374, BroccoCress®, Hi-0), optimal ITC formation was observed, whereas around pH 6 ITC formation was lowest (Figs 2A, 3A and 4A). This low level of ITC formation is probably due to the pH-dependency of the activity of the respective ESP since the optimal pH for ESP from *Brassica napus* and *Crambe abyssinica* was reported to be at pH 6^52^,^56^. Therefore, at higher or lower pH values, the ESP’s ability to catalyse EPT and nitrile formation will decrease and more ITC will be formed. However, no effect of pH was observed for the formation of ITC and EPT in the *A. thaliana* accession Bur-0. This was probably due to the very high constitutive ESP activity compared with the other *Brassica* plants (Table 1). Thus, a slight decrease in ESP activity due to the change in pH possibly did not affect the breakdown product outcome of the reaction. On the other hand, the *A. thaliana* Hi-0 had very low ESP activity, and 2Prop-ITC was always the main breakdown product. However, Hi-0 followed the trend of the other investigated *Brassica* plants and formed more ITC at basic and acidic pH. In contrast to the other plants with higher ESP activity, EPT concentration was much less affected by the pH value, whereas the nitrile concentration was. Thus, degradation of 2Prop-GLS in Hi-0 (Fig. 3) was more similar to degradation of 4MSOB-GLS in BroccoCress® (Fig. 4). Usually, if no ESP were present/active, it would be expected that at low pH, nitrile formation would be favoured and that with increasing pH, ITC formation would increase^18^. Thus, a possible explanation could be that the nitriles of Hi-0 derive from NSPs present in *A. thaliana*. In this context, especially NSP1 was reported to be responsible for nitrile formation in *A. thaliana*^17^,^22^,^24^. Thus, as the pH optimum of NSP1 is similar to that of ESP^57^, presence of NSPs could also explain the high formation of nitriles at pH 6.4 – a process that decreases at high and low pH.

Although much less than the effect of pH, temperature also influenced the enzymatic hydrolysis of GLS in these Brassicaceae plants, but with less stringency (Figs 2, 3 and 4, Table 2). This might be explained by different temperature optima of the different ESPs of the investigated plants of the Brassicaceae family. Burow *et al*.^51^ described for ESP in *A. thaliana* an increased absolute and relative nitrile formation at lower temperatures (0–20 °C), although myrosinase activity was reduced^51^. However, in the present study, the *A. thaliana* accession Bur-0 showed an inverse effect, and at acidic pH, ITC and nitrile formation was highest at 37 °C, while EPT formation was decreased at 37 °C, thereby suggesting an ecotype-dependent effect due to genotypic climate adaptation. Moreover, in addition to ESP, the enzymatic activity of myrosinase will be influenced by the pH and temperature. While myrosinase in broccoli was characterized to have the highest activity at pH 6.5–7^58^, myrosinase in Brussels sprouts has an additional optimum at pH 8^59^. Furthermore, the temperature optimum for myrosinase activity in broccoli and Brussels sprouts was reported to be 30 °C^58^ and 50 °C^59^, respectively. Thus, if myrosinase activity increases due to changes in pH and/or temperature, while ESP activity is not affected, the ratio of GLS-aglucon to ESP would also increase and this would lead to lower EPT and higher ITC formation^51^.

Another influencing factor on ITC formation might be the concentration of the plant material, e.g. the dilution. Therefore, dilution with water, saliva buffer and myrosinase-inactivated plant material was studied and the results were then modelled to obtain information on the reaction kinetics of the GLS degradation. With all diluents types and in all investigated *Brassica* plants, the dilution increased ITC formation. This finding could be explained by the reaction mechanisms (compare with Fig. 1). In detail, dilution will result in a decrease both in myrosinase and ESP concentration, and thereby lowering the effective activity of these two enzymes. In effect, the GLS will be degraded by the myrosinase more slowly to form the instable aglucon. While the kinetics of the non-spontaneous degradation should not be much affected, the probability of the ESP encountering the aglucon will be decreased due to the dilution. Therefore, the spontaneous degradation leading to ITC formation will be promoted.

Kinetic modelling of the hydrolysis reaction revealed that the GLS in the plants responded differently to the diluents types. While in water the reaction to form ITC was slowest (lowest k_4_ or x_4_), ITC formation was increased in saliva buffer and when diluted with the myrosinase-inactivated plant homogenate (Table 3). These findings can be explained with the increase of pH value caused by the saliva buffer addition (pH 7) as well as with the increasing GLS concentration caused by the addition of myrosinase-inactivated plant tissue which still contained GLS. In this case, the ESP concentration per GLS was much more decreased upon dilution with myrosinase-inactivated plant material compared to the dilution with water (or saliva buffer), and thus, the dilution effect was multiplied. However, while the models on the optimization of ITC formation by 2Prop-GLS hydrolysis in white cabbage sprouts and 4MTB-GLS hydrolysis in BroccoCress® are similar, dilution models within one *Brassica* species, namely BroccoCress®, can differ such as between 4-MTB- and 4MSOB-GLS hydrolyses.

While the effect of water and myrosinase-inactivated plant material was similar (Table 3), addition of neutral saliva buffer had a stronger effect on 4MSOB-ITC than on 4MTB-ITC optimization. Moreover, in the experiment on pH and temperature, 4MSOB-ITC formation in BroccoCress® was more increased by the basic pH compared to 4MTB-ITC and 4MSOB-ITC was even favoured above the corresponding nitrile. Further, it should be considered that ESP activity is specific to the GLS structure^51^. Therefore, it is suspected that the susceptibility of the substrate-specific ESP activity towards a change in pH is also structure-specific, thus affecting the hydrolysis of different GLS differently.

## Conclusion

The hydrolysis of GLS is crucial to elicit the health beneficial effects linked to GLS breakdown compounds, as mainly ITCs exert these effects. The present study reveals how ITC formation can be optimized by selection of optimal pH values and temperature or by diluting the vegetable matrix in typical domestic preparation procedures. As the pH value greatly affects ITC formation, acidic (pH 4) and alkaline (pH 8) pH are optimal to obtain high ITC concentrations. Temperature also influences ITC formation; however, the effect is weaker and is also dependent on the *Brassica* species. For example, ITC formation in *B. oleracea* var. *italica* BroccoCress® increased with temperature and 37 °C was found to be optimal. When the myrosinase and ESP are diluted, ITC formation will be favoured, and thus, dilution also represents a valuable strategy to increase ITC formation. Of note is that the dilution with cooked, and therefore, myrosinase-inactivated plant material resulted in the highest ITC concentrations. The degree of ITC optimization depend on the GLS-structure.

This study shows that promoting higher consumption of vegetables may not be the most efficient way of increasing the intake of the healthy compounds such as ITC. Domestic processing and handling methods as well as chewing have a much stronger effect on ITC formation, and thus, their intake. Based on the results of the present study, general recommendations for food preparation can be given to optimize ITC formation, thereby enhancing health-beneficial effects attributed to them. In detail, *Brassica* vegetables should be prepared either after adding acid, such as for cabbage salad with the addition of lemon juice or vinaigrette, or prepared while using natron (NaHCO_3_), as is common for (blue) red cabbage. Moreover, if vegetables are homogenized, it is advisable to add water or other diluents as this will also result in optimal ITC content. In conclusion, this study provides new insights into the future design of food products rich in health-promoting ITCs.

## Methods

### Chemicals and buffers

Acetic acid (≥99.5%), 2Prop-GLS (≥99%), l-ascorbic acid (≥99%), benzonitrile (≥99.9%), d/l-dithiothreitol, FeSO_4_(H_2_O)_7_ (≥99%), myrosinase (thioglucosidase from *Sinapis alba* seeds; ≥100 units/g) and NaHCO_3_ (≥99.5%) were purchased from Sigma-Aldrich Chemie GmbH, (Steinheim, Germany). 4-Hydroxybenzyl GLS (≥99%) was purchased from Carl Roth GmbH, Karlsruhe, Germany and NaCH_3_COO(H_2_O)_3_ (≥99.5%) was purchased from Merck (Darmstadt, Germany), respectively. Simulated salivary fluid (saliva buffer) was prepared according to^60^ (pH 7) using chemicals from Merck (Darmstadt, Germany) (KCl, KH_2_PO_4_, MgCl_2_(H_2_O)_6_, CaCl_2_(H_2_O)_2_) and Sigma-Aldrich Chemie GmbH, (Steinheim, Germany) (α-amylase. NaHCO_3_, HCl (37%), (NH_4_)_2_CO_3_ (≥30%, NH_3_ basis)) with a purity of ≥99% if not stated otherwise. All solvents were of LC-MS grade and water was of Milli-Q quality.

### Plant material

Two *A. thaliana* accessions Bur-0 and Hi-0 (kindly provided by L. Westphal, Leibniz Institute of Plant Biochemistry, Halle, Germany) having a contrasting GLS breakdown product profile^40^ and two *B. rapa* accessions, namely BR215 (B. *rapa* ssp. *rapa* L. f. Komatsuna) and BR374 (*B. rapa* ssp. *oleifera* (DC.) Metzger f. annua), both part of the Vavilov Research Institute of Plant Industry (VIR) *B. rapa* core collection^61^ (kindly provided by Anna Artemyeva, VIR, St. Petersburg, Russia), were selected for the experiments and grown in a climate chamber.

Seeds were germinated in soil (“Einheitserde Classic”, medium structure, pH value 5.9, Einheitserde Werkverband e.V., Germany), seedlings (10 days old) were transplanted into single pots filled with soil and plants were grown in a climate chamber at controlled light (*A. thaliana*: 8 h photoperiod, 320 μmol·m^−2^·s^−1^; *B. rapa*: 12 h photoperiod, 400 μmol·m^−2^·s^−1^) and a temperature regime with a light temperature of 20 °C (*A. thaliana*) or 22 °C *(B. rapa*) and a dark temperature of 18 °C (*A. thaliana*) or 20 °C (*B. rapa*) as well as defined humidity (70% for *A. thaliana*; 65% for *B. rapa*). Water was given as needed. After 5 weeks (*B. rapa*) or 6 weeks (*A. thaliana*), leaf tissue was harvested and immediately subjected to experiments.

Further, sprouts of two *B. oleracea* varieties were used in the experiments: BroccoCress® sprouts (*B. oleracea* var. *italica*) were kindly provided by Koppert Cress, Monster, The Netherlands and white cabbage sprouts (*B. oleracea* convar. *capitata* var. *alba* cultivar Langedijker Bewaar) were grown on soil (Lentse Potgrond, Hortimea groep, Lent, The Netherlands) for 10 days in a lab at room temperature. Water was given as needed for optimal sprout growth.

### Harvest and experimental design

The experiments on the effects of pH value and temperature as well as sampling for all other analyses were performed on the first day, while the experiment on dilution was performed on the day after. At first harvest day, leaf tissue of 20 *B. rapa* (without midrip) or 90 *A. thaliana* plants or of several trays of sprouts was harvested and mixed. An aliquot of each mixed tissue was immediately deep-frozen at −50 °C and lyophilized. The lyophilized material was kept at −20 °C until further analysis. From the edible plants (*B. rapa* and *B. oleracea*), another aliquot of the plant tissue was subjected to microwave treatment, in order to inactivate the myrosinase^62^. Evaporated water was then resupplied and the inactivated material was homogenized using a mixer mill at a frequency of 30 Hz (Retsch MM 400, Retsch GmbH, Haan, Germany). From this juice, an aliquot was lyophilized and the rest was used for the dilution experiments as inactivated plant material and stored at −20 °C until the next day.

The rest of the fresh material was used for the experiments on the effect of pH value, temperature and dilution on the breakdown of GLS. For this purpose, leaf tissue of *A. thaliana* and *B. rapa* was cut into 0.5 cm^2^ big pieces and 250 mg of this tissue or of the sprouts were weighted into a 20 mL Polyvial® (Zinsser Analytic GmbH, Frankfurt, Germany). For the experiment on the effect of pH value and temperature, 500 μL of either 0.1 M acetic acid (for pH 4), 500 μL of water (pH 6) or 500 μL of 0.1 M NaHCO_3_ (pH 8) were added and the vials with the plant material/buffer mixture were acclimatized for 10 min either to 4, 22 or 37 °C. The plant tissue was then homogenized using the mixer mill at 4, 22 or 37 °C within 1 min at a frequency of 30 Hz. After which, plant tissue was incubated at the already mentioned temperatures for another 10 min. These 10 min should have been sufficient for a complete breakdown of the GLS, as demonstrated in a pre-experiment with fresh *B. rapa* tissue for which after 1 min of hydrolysis at 0 °C, the degradation reaction was already complete.

For the experiment on dilution on the second day of harvest, 10 *B. rapa* (without midrip) or 30 *A. thaliana plants* were harvested, cut into small pieces and then mixed as described above. To 250 mg of freshly harvested plant material in the Polyvial®, 250 μL, 500 μL, 2 mL or 4 mL (only in experiment with water) of water, saliva buffer (see above) or the inactivated plant material described above were added. The plant tissue was then acclimatized and homogenized at 22 °C (water, inactivated plant material) or 37 °C (saliva buffer) and incubated as described above. The tissues were finally subjected to analysis of the formed degradation products. Each experiment was performed in triplicate.

### Analysis of glucosinolates

Plant tissues used for the experiments as well as the inactivated plant material were characterized for their GLS content. For this purpose, the method of Witzel *et al*.^40^ was adapted. Briefly, 20 mg of lyophilized and ground plant tissue were extracted using 70% methanol in the presence of 0.5 μmol 4-hydroxybenzyl GLS as an internal standard. The combined extracts were loaded onto DEAE-Sephadex A-25 ion-exchanger columns, desulphated using aryl sulfatase and then desulpho-GLSs were eluted with water. Analysis of desulpho-GLSs was performed as described recently by Witzel *et al*.^40^ using an UHPLC Agilent 1290 Infinity System (Agilent Technologies, Böblingen, Germany) and a gradient of water and acetonitrile, and then quantified at 229 nm via the internal standard. Each material was analysed with two technical replicates.

### Determination of glucosinolate breakdown products after incubation

The quantification of the enzymatically formed GLS breakdown products was performed according to Witzel *et al*.^40^. After the experiment on pH and temperature, all the plant material was quantitatively transferred into a 10 mL centrifuge tube with a lid. For the experiment on dilution and for the inactivated plant material, 250 mg of the tissue were weighted into a centrifuge tube. The GLS hydrolysis products were then extracted and analysed as reported previously^40^. Briefly, hydrolysis products were extracted using methylene chloride (Actu-All Chemicals b.v., Oss, The Netherlands) in the presence of 0.2 μmol of the internal standard benzonitrile. Extracts were then dried using anhydrous sodium sulphate, concentrated under nitrogen gas flow to 300 μL, transferred into a vial and analysed by GC-MS according to Witzel *et al*.^40^ using a SGE BPX5 GC-MS column (30 m × 0.25 mm × 0.25 μm) (VWR International GmbH, Darmstadt, Germany). Each analysis consisted of tree independent replicates.

### Analysis of myrosinase activity

The myrosinase enzyme was extracted according to Oliviero *et al*.^30^ with some modifications. In detail, 30 mL of potassium phosphate buffer (50 mM, pH 7.0) was added to 0.03 g of dry plant tissue and stirred overnight at 20 °C. The potassium phosphate buffer solution was then centrifuged at 2670 g for 10 min and the supernatant was filtered (folded filters Grade 595 1/2–4–7 μm, Whatman). Finally, the supernatant was filtered through cut-off filter tubes (Amicon Ultra-4 cut-off 30 kDa, Millipore) to concentrate the myrosinase. The residue was dissolved again using 0.5 mL of the potassium phosphate buffer and the myrosinase activity was determined according to a coupled enzymatic procedure described by Van Eylen *et al*.^63^, with some modifications. In this assay, d-glucose, which is formed in the myrosinase catalysed hydrolysis reaction of 2-propenyl GLS, is measured by transforming NADP^+^ to NADPH (d-Glucose HK assay kit, K-GLUHK-110A, Megazyme). The reaction mixture consisted of 0.9 mL of a watery solution containing 0.05 g/L MgCl_2_ and 1 g/L l-ascorbic acid (vitamin C), 50 μL of the extracted myrosinase solution, 50 μL of test kit solution Bottle1 (imidazole buffer, MgCl_2_ and sodium azide), 50 μL of test solution Bottle2 (NADP^+^, ATP), 5 μL of test solution Bottle3 (hexokinase (300 U/mL) plus glucose-6-phosphate dehydrogenase (400 U/mL) and 50 μL of a 30 mg/mL solution of 2-propenyl GLS. The formation of NADPH was followed by a spectrophotometer (Cary UV 50, Varian, Bergen op Zoom, The Netherlands) at 340 nm for 7 min. The activity was determined based on the slope of the linear part of the curve of absorbance versus reaction time. To quantify the myrosinase activity, a calibration curve was established with an external standard myrosinase in the phosphate buffer solution with dilutions of ranging from 0.01 to 1.0 U/mL (R^2^ = 0.975) by following the same procedure as for the sample analysis. Activity was expressed as U/g dry weight.

### Determination of epithiospecifier protein activity

To compare the ESP activities of the different plant materials, the protocol of Matusheski *et al*.^28^ was adapted: To 20 mg of lyophilized plant material, 380 μL of water were added, the sample thoroughly vortexed and then shaken for 10 min on ice. Samples were then centrifuged (16000 g, 4 °C, 20 min) and the supernatant (ESP extract) was used for the determination of the ESP activity. The ESP activity assay was performed according to Matusheski *et al*.^28^, except allyl GLS was used instead of 2(*S*)-hydroxy-3-butenylglucosinolate. Briefly, 50 μL of ESP extract, 350 μL of a 50 mM sodium acetate buffer (pH 5.5) containing 1 mM dithiothreitol and 1 mM of FeSO_4_, 50 μL of 0.5 U/mL myrosinase and 50 μL of 2Prop-GLS (5 mg/mL) were mixed and incubated for 1 h at room temperature. Hydrolysis products of 2Prop-GLS [CETP, 2Prop-ITC and 2Prop-CN)] were extracted and quantified according to the protocol described above. For samples that already contained allyl GLS (Bur-0, Hi-0, cabbage sprouts), blanks without allyl GLS were also prepared (with 50 μL of water added instead) and the small concentrations determined in these samples were used to correct the values of the positive controls. ESP activity was expressed as the % of EPT = [CETP]/([CETP] + [2Prop-ITC] + [2Prop-CN]) * 100%. Each analysis consisted of three independent replicates.

### Quantification of l-ascorbic acid content

Vitamin C (l-ascorbic acid) content was determined photometrically with the ENZYTEC^TM^ l-Ascorbic Acid assay (R-Biopharm AG, Darmstadt, Germany). The extraction of l-ascorbic acid was based on the manufacturer’s instructions. Fresh leaf material (250 mg) was weighted into 2 mL reaction tubes and 1 mL of 1.5% meta-phosphoric acid (pH 3.5) and 4 steel balls (4 mm) were added. The plant material was homogenized using the mixer mill for 4 min at a frequency of 30 Hz. After 20 min centrifugation at 4 °C (13000 rpm), the supernatant was used for measurement according to the manufacturer’s instructions. For quantification, an external calibration curve of l-ascorbic acid was used. Each analysis consisted of four biological replicates.

### Modelling of the data

To correlate the effect of pH and temperature on the enzymatic degradation of GLS in the different plants, response surface modelling was performed using Design Expert software, version 10.0.2.0. (Stat-Ease, Inc., Minneapolis, MN, USA). For the modelling, relative values were used: Prior to modelling, the percentage of the respective ITC, nitrile and EPT relative to the sum of the breakdown products formed from the parent GLS was calculated for each sample. Kinetic models for the effect of dilution on the enzymatic degradation of GLS were developed using Microsoft Office 2010 Excel software with macropack × (ref). The reactions shown in Fig. 1 were used as a basis to calculate equations for the formation of the respective breakdown products (see final equations in the results part). The relative values for the breakdown products formed under the respective conditions were used for the calculation. With help of the Solver Add-In, the sum of the squares from the percentage of measured ITC formation and from the modelled values was minimized by optimization of the constants. During modelling, the kinetic model highlighted that k_3_ = 0. Therefore, equations could be simplified and either k_1_ and k_4_ or x_1_ = k_1_/k_2_ and x_4_ = k_4_/k_2_ could be calculated.

## Additional Information

**How to cite this article:** Hanschen, F. S. *et al*. Optimizing isothiocyanate formation during enzymatic glucosinolate breakdown by adjusting pH value, temperature and dilution in *Brassica* vegetables and *Arabidopsis thaliana. Sci. Rep.* **7**, 40807; doi: 10.1038/srep40807 (2017).

**Publisher's note:** Springer Nature remains neutral with regard to jurisdictional claims in published maps and institutional affiliations.

## Supplementary Material

Supplementary Information

## Figures and Tables

**Table 1:** Myrosinase activity (MYR) [U/g fresh weight], ESP activity [%CEPT], vitamin C content [mg/100 g fresh weight] and total GLS in Brassicaceae species used for the studies.

Assay and Ratios	*Brassica rapa* 215			*Brassica rapa* 374			*Arabidopsis thaliana* Bur-O			*Arabidopsis thaliana* Hi-O			*Brassica oleracea* var. *italica* BroccoCress®			*Brassica oleracea* var. *capitata* white cabbage		
Myrosinase activity (MYR) [U/g fresh weight]	32.98	±	2.34	27.03	±	1.07	544.42	±	1.84	88.98	±	6.50	331.22	±	46.61	2.41	±	0.33
ESP activity [%CEPT]	4.93	±	1.19	5.70	±	0.32	49.79	±	1.83	1.26	±	0.30	32.96	±	1.29	3.72	±	0.18
Vitamin C [mg/100 g fresh weight]	31.53	±	6.84	2.29	±	0.72	5.57	±	1.16	6.30	±	1.05	0.59	±	0.25			
ESP/MYR	0.15			0.21			0.09			0.01			0.10			1.55		
Total GLS [μmol/g fresh weight]	0.67	±	0.01	1.63	±	0.05	6.93	±	0.04	5.87	±	0.01	7.69	±	0.02	2.79	±	0.02
MYR/GLS	49.5			16.6			78.5			15.2			43.1			0.9		
ESP/GLS	7.40			3.51			7.18			0.21			4.29			1.33		

ESP activity in [%CEPT] is defined as the percentage of CETP of all breakdown products from 2Prop-GLS formed in the assay upon hydrolysis.

**Table 2:** Response surface models for the influence of pH value and temperature on the enzymatic degradation of GLS in different Brassicaceae species.

	*Brassica rapa* 215			*Brassica rapa* 374			*Brassica oleracea* var. *italica* BroccoCress®		*Arabidopsis thaliana* Hi-0			*Arabidopsis thaliana* Bur-0	
Degradation product	3But-ITC	3But-CN	CETB	3But-ITC	3But-CN	CETB	4MSOB-CN	4MSOB-ITC	2Prop-ITC	2Prop-CN	CETP	2Prop-CN	3But-CN
Model	quadratic	reduced quadratic	reduced quadratic	reduced quadratic	reduced quadratic	quadratic	reduced quadratic	reduced quadratic	reduced quadratic	reduced quadratic	2FI model	reduced quadratic	linear
Transformation	square root	square root			natural log					natural log	natural log	inverse	natural log
Model	****	****	****	^0^*	**	*	****	****	****	****	****	****	****
Intercept	+2.97	+1.45	+82.85	+31.27	+1.70	+64.06	+42.47	+57.53	+69.70	+3.32	−1.81	0.38	8.18
A-pH	+9.74****		−52.90*	+39.68^0^*	+1.45*	−48.51*	+126.86****	−126.86****	+58.83****	−2.59****	−4.08****		−9.49****
B-T	−1.86***	+1.94*		−17.14*		+16.38*	+22.63****	−22.63****	+5.70**	−0.28***	−0.28*	−0.16****	+5.42****
AB	+5.95***		−49.15*		−1.82**	+23.72^n.s^			+19.02**	−1.01***	−2.70****		
A^2^	+40.57****	+23.45****	−440.11****	+183.07*	+6.42 **	−237.72**	+335.53****	−335.53****	+192.48****	−7.89****		−1.09****	
B^2^	−2.78 **	+3.55*		−27.61^0^*		+23.49^0^*						−0.11***	
Lack of Fit	n.s.	n.s.	n.s.	n.s.	n.s.	n.s.	n.s.	n.s.	n.s.	n.s.	n.s.	n.s.	n.s.

Asterisks indicate the level of significance: *p ≤ 0.1; *p ≤ 0.05; **p ≤ 0.01; ***p ≤ 0.001; ****p ≤ 0.0001; n.s. not significant (p ≥ 0.05). Abbreviations are according to the list of abbreviations.

**Table 3:** Models for the effect of dilution on the enzymatic degradation of GLS in *Brassica rapa* and *Brassica oleracea* species.

Plant	Dilutant	Glucosinolate	Calculated model parameters			
			SS ± SD	x_1_ ± SD	x_4_ ± SD	CC
*Brassica rapa* 215	Water	3-Butenyl	704.6 ± 4.6	29.72 ± 11.09	0.766 ± 0.304	0.984
	Saliva buffer	3-Butenyl	1740.2 ± 8.3	288.5 ± 2302.5	18.08 ± 144.90	1.000
	Inactivated plant	3-Butenyl	397.0 ± 4.0	12.60 ± 2.16	0.473 ± 0.098	0.922
*Brassica rapa* 374	Water	3-Butenyl	4039.9 ± 10.9	9.91 ± 3.78	0.603 ± 0.250	0.952
	Saliva buffer	3-Butenyl	1485.3 ± 7.7	9.37 ± 3.13	1.31 ± 0.46	0.965
*B. oleracea* var. *capitata,* white cabbage	Water	2-Propenyl	718.1 ± 4.6	6.27 ± 0.59	0.126 ± 0.018	0.770
	Saliva buffer	2-Propenyl	1429.0 ± 7.6	3.17 ± 0.46	0.486 ± 0.082	0.817
	Inactivated plant	2-Propenyl	2074.7 ± 9.1	3.56 ± 0.72	0.691 ± 0.153	0.868
*B. oleracea* var. *italica*, BroccoCress®			SS ± SD	k_2_ ± SD	k_4_ ± SD	CC
	Water	4-(Methylthio)butyl	492.6 ± 4.7	1.02 ± 0.02	0.022 ± 0.002	−0.399
		4-(Methylsulphinyl)butyl	1250.7 ± 7.5	1.06 ± 0.04	0.068 ± 0.005	−0.265
	Saliva buffer	4-(Methylthio)butyl	265.9 ± 4.1	1.02 ± 0.02	0.089 ± 0.005	−0.337
		4-(Methylsulphinyl)butyl	944.1 ± 7.7	1.03 ± 0.13	0.840 ± 0.086	*0.350*
	Inactivated plant	4-(Methylthio)butyl	846.0 ± 7.3	0.99 ± 0.05	0.150 ± 0.012	−0.259
		4-(Methylsulphinyl)butyl	1495.7 ± 9.7	1.08 ± 0.08	0.175 ± 0.019	−0.169

The reaction rate coefficients k_1_–k_4_ are the ones indicated for the reactions in Fig. 1 with x1 = k1/k2 and x4 = k4/k2. SS: Sum of squares (SUMMEXMY2); SD: standard deviation; CC: correlation coefficient between x_1_/x_4_ or k_1_/k_4_.
