# Supplementary material for: Optimizing isothiocyanate formation during enzymatic glucosinolate breakdown by adjusting pH value, temperature and dilution in Brassica vegetables and Arabidopsis thaliana
Source: Sci Rep. 2017 Jan 17;7:40807. doi: 10.1038/srep40807 (PMC5240131; doi:10.1038/srep40807)

Optimizing isothiocyanate formation during enzymatic glucosinolate breakdown by adjusting pH value, temperature and dilution in *Brassica* vegetables and *Arabidopsis thaliana*

Franziska S. Hanschen[1]*, Rebecca Klopsch[1], Teresa Oliviero[2], Monika Schreiner[1], Ruud Verkerk[2] and Matthijs Dekker[2]

[1]Department of Plant Quality, Leibniz Institute of Vegetable and Ornamental Crops, Theodor-Echtermeyer-Weg 1, 14979 Grossbeeren, Germany
[2]Food Quality and Design Group, Wageningen University, PO Box 17, 6700 AA Wageningen, The Netherlands

*Corresponding author:
Franziska S. Hanschen
Leibniz Institute of Vegetable and Ornamental Crops, Theodor-Echtermeyer-Weg 1, 14979 Grossbeeren, Germany
Tel: 0049-33701-78241
Fax: 0049-33701-55391
Email: hanschen@igzev.de

**Glucosinolates (GLS) in µmol/g fresh weight**	***Brassica rapa* 215**		**Inactivated *Brassica rapa* 215**		***Brassica rapa* 374**		**Inactivated *Brassica rapa* 374**		***Arabidopsis thaliana* Bur-0**		***Arabidopsis thaliana* Hi-0**		***Brassica oleracea* BroccoCress®**		**Inactivated *Brassica oleracea* BroccoCress®**		***Brassica oleracea* white cabbage**		**Inactivated *Brassica oleracea* white cabbage**	
	**MW**	SD	**MW**	SD	**MW**	SD	**MW**	SD	**MW**	SD	**MW**	SD	**MW**	SD	**MW**	SD	**MW**	SD	**MW**	SD
**2-propenyl (2Prop)**	**0.000**	0.000	**0.000**	0.000	**0.000**	0.000	**0.000**	0.000	**1.806**	0.019	**5.088**	0.003	**0.000**	0.000	**0.000**	0.000	**1.231**	0.018	**2.357**	0.086
**3-butenyl (3But)**	**0.296**	0.003	**0.102**	0.001	**1.126**	0.034	**0.676**	0.024	**2.910**	0.011	**0.036**	0.000	**0.020**	0.000	**0.003**	0.001	**0.033**	0.003	**0.048**	0.001
**4-pentenyl**	**0.194**	0.006	**0.025**	0.000	**0.391**	0.012	**0.074**	0.003	**0.073**	0.005	**0.000**	0.000	**0.000**	0.000	**0.000**	0.000	**0.000**	0.000	**0.000**	0.000
***R*-2-OH-3-butenyl**	**0.053**	0.001	**0.030**	0.002	**0.000**	0.000	**0.000**	0.000	**0.198**	0.005	**0.000**	0.000	**0.011**	0.001	**0.000**	0.000	**0.428**	0.025	**0.701**	0.027
***S*-2-OH-3-butenyl**	**0.000**	0.000	**0.000**	0.000	**0.000**	0.000	**0.000**	0.000	**0.355**	0.001	**0.000**	0.000	**0.000**	0.000	**0.000**	0.000	**0.000**	0.000	**0.000**	0.000
**3-(methylthio)propyl**	**0.000**	0.000	**0.000**	0.000	**0.000**	0.000	**0.000**	0.000	**0.046**	0.001	**0.000**	0.000	**0.007**	0.001	**0.000**	0.000	**0.024**	0.001	**0.098**	0.015
**4-(methylthio)butyl**	**0.000**	0.000	**0.000**	0.000	**0.000**	0.000	**0.000**	0.000	**0.000**	0.000	**0.000**	0.000	**1.119**	0.045	**1.114**	0.001	**0.014**	0.002	**0.023**	0.001
**3-(methylsulphinyl)propyl**	**0.000**	0.000	**0.000**	0.000	**0.000**	0.000	**0.000**	0.000	**0.000**	0.000	**0.000**	0.000	**0.163**	0.001	**0.151**	0.002	**0.587**	0.012	**1.467**	0.065
**4-(methylsulphinyl)butyl**	**0.066**	0.025	**0.024**	0.011	**0.010**	0.002	**0.033**	0.010	**0.038**	0.014	**0.000**	0.000	**5.856**	0.078	**5.923**	0.134	**0.240**	0.011	**0.486**	0.021
**7-(methylsulphinyl)heptyl**	**0.000**	0.000	**0.000**	0.000	**0.000**	0.000	**0.000**	0.000	**0.196**	0.001	**0.034**	0.000	**0.000**	0.000	**0.000**	0.000	**0.000**	0.000	**0.000**	0.000
**8-(methylsulphinyl)octyl**	**0.000**	0.000	**0.000**	0.000	**0.000**	0.000	**0.000**	0.000	**0.925**	0.017	**0.477**	0.014	**0.000**	0.000	**0.000**	0.000	**0.000**	0.000	**0.000**	0.000
**2-phenylethyl**	**0.012**	0.002	**0.004**	0.000	**0.031**	0.002	**0.011**	0.000	**0.017**	0.004	**0.000**	0.004	**0.019**	0.000	**0.012**	0.000	**0.000**	0.000	**0.001**	0.000
**3-indolylmethyl**	**0.080**	0.002	**0.034**	0.001	**0.023**	0.002	**0.011**	0.001	**0.252**	0.004	**0.164**	0.004	**0.186**	0.005	**0.165**	0.001	**0.093**	0.004	**0.154**	0.005
**1-methoxy-3-indolylmethyl**	**0.005**	0.002	**0.003**	0.000	**0.028**	0.002	**0.010**	0.001	**0.044**	0.000	**0.042**	0.001	**0.047**	0.001	**0.052**	0.003	**0.016**	0.000	**0.023**	0.001
**4-hydroxy-3-indolylmethyl**	**0.006**	0.000	**0.003**	0.001	**0.004**	0.001	**0.003**	0.000	**0.007**	0.001	**0.005**	0.001	**0.147**	0.001	**0.121**	0.001	**0.112**	0.008	**0.194**	0.013
**4-methoxy-3-indolylmethyl**	**0.021**	0.001	**0.015**	0.000	**0.014**	0.000	**0.006**	0.000	**0.065**	0.004	**0.026**	0.000	**0.113**	0.003	**0.102**	0.001	**0.012**	0.001	**0.007**	0.000
**Total GLS**	**0.666**	0.011	**0.240**	0.011	**1.627**	0.054	**0.824**	0.015	**6.933**	0.041	**5.872**	0.007	**7.687**	0.020	**7.643**	0.135	**2.790**	0.024	**5.559**	0.235

Influence of temperature and pH value

µmol/g fresh weight

Brassica rapa 215	**4°C, pH 4.2**		**4°C, pH 6.3**		**4°C, pH 8.3**	4	**22°C, pH 4.2**		**22°C, pH 6.3**		**22°C, pH 8.3**		**37°C, pH 4.2**		**37°C, pH 6.3**		**37°C, pH 8.3**	
3-butenyl CN	**0.0185 ±**	0.0093	**0.0034 ±**	0.0010	**0.0097 ±**	0.0029	**0.0064 ±**	0.0055	**0.0024 ±**	0.0013	**0.0017 ±**	0.0019	**0.0105 ±**	0.0084	**0.0130 ±**	0.0206	**0.0061 ±**	0.0064
3-butenyl ITC	**0.0223 ±**	0.0019	**0.0067 ±**	0.0043	**0.0162 ±**	0.0014	**0.0076 ±**	0.0050	**0.0056 ±**	0.0028	**0.0146 ±**	0.0038	**0.0020 ±**	0.0006	**0.0000 ±**	0.0000	**0.0104 ±**	0.0008
1-cyano-3,4-epithiobutane	**0.0052 ±**	0.0011	**0.0687 ±**	0.0202	**0.0507 ±**	0.0051	**0.0101 ±**	0.0117	**0.0571 ±**	0.0210	**0.0241 ±**	0.0151	**0.0081 ±**	0.0037	**0.0182 ±**	0.0026	**0.0200 ±**	0.0042
4-pentenyl ITC	**0.0506 ±**	0.0135	**0.0015 ±**	0.0017	**0.0032 ±**	0.0005	**0.0294 ±**	0.0477	**0.0020 ±**	0.0006	**0.0032 ±**	0.0006	**0.0000 ±**	0.0000	**0.0006 ±**	0.0010	**0.0015 ±**	0.0007
1-cyano-4,5-epithiopentane	**0.0032 ±**	0.0004	**0.0156 ±**	0.0045	**0.0066 ±**	0.0005	**0.0031 ±**	0.0027	**0.0095 ±**	0.0023	**0.0047 ±**	0.0006	**0.0011 ±**	0.0019	**0.0038 ±**	0.0013	**0.0037 ±**	0.0007
2-phenylethyl CN	**0.0081 ±**	0.0030	**0.0040 ±**	0.0017	**0.0017 ±**	0.0004	**0.0053 ±**	0.0055	**0.0034 ±**	0.0014	**0.0016 ±**	0.0003	**0.0013 ±**	0.0001	**0.0013 ±**	0.0003	**0.0015 ±**	0.0003
2-phenylethyl ITC	**0.0024 ±**	0.0006	**0.0011 ±**	0.0001	**0.0014 ±**	0.0012	**0.0014 ±**	0.0005	**0.0015 ±**	0.0003	**0.0008 ±**	0.0001	**0.0014 ±**	0.0002	**0.0018 ±**	0.0004	**0.0004 ±**	0.0002

Brassica rapa 374	**4°C, pH 4.2**		**4°C, pH 6.3**		**4°C, pH 8.3**	4	**22°C, pH 4.2**		**22°C, pH 6.3**		**22°C, pH 8.3**		**37°C, pH 4.2**		**37°C, pH 6.3**		**37°C, pH 8.3**	
3-butenyl CN	**0.1030 ±**	0.0575	**0.0697 ±**	0.0071	**0.0477 ±**	0.0141	**0.0568 ±**	0.0437	**0.1249 ±**	0.0558	**0.0808 ±**	0.0447	**0.0889 ±**	0.0503	**0.1762 ±**	0.0913	**0.0690 ±**	0.0662
3-butenyl ITC	**0.3106 ±**	0.0869	**0.5477 ±**	0.1482	**0.6467 ±**	0.2295	**0.3029 ±**	0.2533	**0.4026 ±**	0.2416	**0.6564 ±**	0.2298	**0.6754 ±**	0.5143	**0.4092 ±**	0.1777	**0.3547 ±**	0.3055
1-cyano-3,4-epithiobutane	**1.6773 ±**	1.2678	**0.9409 ±**	0.1564	**0.7627 ±**	0.3608	**1.0321 ±**	1.1254	**1.4932 ±**	1.0723	**0.8237 ±**	0.3709	**0.9370 ±**	0.2860	**1.7951 ±**	0.8852	**0.6017 ±**	0.5355
4-pentenyl ITC	**0.0679 ±**	0.0691	**0.0577 ±**	0.0345	**0.1572 ±**	0.0748	**0.0727 ±**	0.1101	**0.0587 ±**	0.0736	**0.0660 ±**	0.0281	**0.0779 ±**	0.0182	**0.1113 ±**	0.1012	**0.0662 ±**	0.0604
1-cyano-4,5-epithiopentane	**0.2010 ±**	0.1791	**0.0601 ±**	0.0424	**0.1380 ±**	0.1081	**0.2040 ±**	0.3283	**0.3083 ±**	0.4979	**0.0710 ±**	0.0453	**0.0724 ±**	0.0325	**0.3807 ±**	0.5503	**0.0443 ±**	0.0451
2-phenylethyl CN	**0.0235 ±**	0.0202	**0.0156 ±**	0.0128	**0.0104 ±**	0.0074	**0.0242 ±**	0.0306	**0.0272 ±**	0.0209	**0.0227 ±**	0.0168	**0.0237 ±**	0.0217	**0.0256 ±**	0.0102	**0.0072 ±**	0.0063
2-phenylethyl ITC	**0.0140 ±**	0.0076	**0.0193 ±**	0.0110	**0.0106 ±**	0.0036	**0.0117 ±**	0.0058	**0.0221 ±**	0.0156	**0.0281 ±**	0.0210	**0.0314 ±**	0.0239	**0.0221 ±**	0.0052	**0.0086 ±**	0.0075

Arabidopsis thaliana Bur-0	**4°C, pH 4.2**		**4°C, pH 6.3**		**4°C, pH 8**		**22°C, pH 4.2**		**22°C, pH 6.3**		**22°C, pH 8**		**37°C, pH 4.2**		**37°C, pH 6.3**		**37°C, pH 8**	
2-propenyl CN	**0.0248 ±**	0.0090	**0.0124 ±**	0.0060	**0.0115 ±**	0.0054	**0.0240 ±**	0.0082	**0.0150 ±**	0.0010	**0.0119 ±**	0.0057	**0.0410 ±**	0.0115	**0.0128 ±**	0.0038	**0.0199 ±**	0.0070
2-propenyl ITC	**0.0183 ±**	0.0038	**0.0272 ±**	0.0092	**0.0242 ±**	0.0081	**0.0222 ±**	0.0074	**0.0367 ±**	0.0076	**0.0317 ±**	0.0177	**0.0572 ±**	0.0079	**0.0179 ±**	0.0046	**0.0332 ±**	0.0088
1-cyano-2,3-epithiopropane	**0.5712 ±**	0.0850	**0.4891 ±**	0.1957	**0.4426 ±**	0.2189	**0.5002 ±**	0.1549	**0.5435 ±**	0.0668	**0.3834 ±**	0.1630	**0.3367 ±**	0.1658	**0.3126 ±**	0.0868	**0.4535 ±**	0.1535
3-butenyl CN	**0.1044 ±**	0.0450	**0.0415 ±**	0.0429	**0.0393 ±**	0.0422	**0.0987 ±**	0.0386	**0.0842 ±**	0.0114	**0.0461 ±**	0.0309	**0.1295 ±**	0.0421	**0.0659 ±**	0.0121	**0.0876 ±**	0.0258
3-butenyl ITC	**0.0263 ±**	0.0068	**0.0435 ±**	0.0227	**0.0369 ±**	0.0185	**0.0271 ±**	0.0129	**0.0557 ±**	0.0183	**0.0438 ±**	0.0276	**0.0668 ±**	0.0194	**0.0165 ±**	0.0049	**0.0381 ±**	0.0161
1-cyano-3,4-epithiobutane	**0.9930 ±**	0.3247	**0.9383 ±**	0.4846	**0.8514 ±**	0.5239	**1.0288 ±**	0.4774	**1.0590 ±**	0.2590	**0.7077 ±**	0.3108	**0.6644 ±**	0.3814	**0.4646 ±**	0.1398	**0.8601 ±**	0.3931
2-hydroxy-3-butenyl CN	**0.0139 ±**	0.0027	**0.0136 ±**	0.0040	**0.0116 ±**	0.0064	**0.0168 ±**	0.0023	**0.0174 ±**	0.0027	**0.0120 ±**	0.0029	**0.0186 ±**	0.0009	**0.0161 ±**	0.0053	**0.0147 ±**	0.0017
1-cyano-2-hydroxy-3,4-epithiobutane	**0.1319 ±**	0.0256	**0.1441 ±**	0.0406	**0.1110 ±**	0.0640	**0.1423 ±**	0.0226	**0.1592 ±**	0.0228	**0.0990 ±**	0.0249	**0.0960 ±**	0.0284	**0.1152 ±**	0.0403	**0.1155 ±**	0.0102
4-pentenyl ITC	**0.0000 ±**	0.0000	**0.0007 ±**	0.0012	**0.0007 ±**	0.0012	**0.0013 ±**	0.0012	**0.0013 ±**	0.0011	**0.0004 ±**	0.0007	**0.0010 ±**	0.0009	**0.0000 ±**	0.0000	**0.0007 ±**	0.0006
1-cyano-4,5-epithiopentane	**0.0297 ±**	0.0049	**0.0267 ±**	0.0161	**0.0212 ±**	0.0171	**0.0300 ±**	0.0182	**0.0279 ±**	0.0121	**0.0159 ±**	0.0057	**0.0146 ±**	0.0088	**0.0112 ±**	0.0049	**0.0198 ±**	0.0104
2-phenylethyl CN	**0.0113 ±**	0.0011	**0.0113 ±**	0.0025	**0.0102 ±**	0.0034	**0.0112 ±**	0.0027	**0.0112 ±**	0.0013	**0.0096 ±**	0.0013	**0.0083 ±**	0.0019	**0.0082 ±**	0.0012	**0.0111 ±**	0.0013
2-phenylethyl ITC	**0.0017 ±**	0.0004	**0.0011 ±**	0.0001	**0.0009 ±**	0.0001	**0.0009 ±**	0.0007	**0.0012 ±**	0.0003	**0.0011 ±**	0.0001	**0.0009 ±**	0.0003	**0.0023 ±**	0.0004	**0.0017 ±**	0.0002
5-(methylthio)pentyl CN	**0.0020 ±**	0.0004	**0.0020 ±**	0.0006	**0.0015 ±**	0.0008	**0.0023 ±**	0.0006	**0.0023 ±**	0.0004	**0.0012 ±**	0.0004	**0.0013 ±**	0.0006	**0.0012 ±**	0.0003	**0.0016 ±**	0.0002
6-(methylthio)hexyl CN	**0.0046 ±**	0.0009	**0.0034 ±**	0.0014	**0.0030 ±**	0.0018	**0.0036 ±**	0.0015	**0.0033 ±**	0.0002	**0.0023 ±**	0.0007	**0.0018 ±**	0.0010	**0.0014 ±**	0.0005	**0.0030 ±**	0.0011
7-(methylthio)heptyl CN	**0.0217 ±**	0.0022	**0.0176 ±**	0.0064	**0.0175 ±**	0.0087	**0.0177 ±**	0.0043	**0.0164 ±**	0.0018	**0.0129 ±**	0.0040	**0.0085 ±**	0.0052	**0.0062 ±**	0.0030	**0.0152 ±**	0.0038
7-(methylthio)heptyl ITC	**0.0017 ±**	0.0007	**0.0024 ±**	0.0034	**0.0035 ±**	0.0021	**0.0031 ±**	0.0032	**0.0055 ±**	0.0017	**0.0027 ±**	0.0027	**0.0039 ±**	0.0024	**0.0058 ±**	0.0001	**0.0026 ±**	0.0031
8-(methylthio)octyl CN	**0.0413 ±**	0.0039	**0.0276 ±**	0.0110	**0.0335 ±**	0.0143	**0.0230 ±**	0.0077	**0.0220 ±**	0.0034	**0.0187 ±**	0.0059	**0.0106 ±**	0.0080	**0.0080 ±**	0.0042	**0.0237 ±**	0.0065
8-(methylthio)octyl ITC	**0.0058 ±**	0.0006	**0.0013 ±**	0.0012	**0.0020 ±**	0.0010	**0.0032 ±**	0.0024	**0.0010 ±**	0.0001	**0.0008 ±**	0.0008	**0.0068 ±**	0.0050	**0.0007 ±**	0.0007	**0.0020 ±**	0.0016
8-(methylsulphinyl)octyl CN	**0.0170 ±**	0.0081	**0.0154 ±**	0.0088	**0.0117 ±**	0.0055	**0.0116 ±**	0.0057	**0.0175 ±**	0.0031	**0.0143 ±**	0.0096	**0.0084 ±**	0.0045	**0.0106 ±**	0.0064	**0.0174 ±**	0.0106
indole-3-acetonitrile	**0.0138 ±**	0.0014	**0.0175 ±**	0.0036	**0.0125 ±**	0.0040	**0.0076 ±**	0.0010	**0.0174 ±**	0.0007	**0.0115 ±**	0.0016	**0.0029 ±**	0.0016	**0.0121 ±**	0.0051	**0.0140 ±**	0.0023

Arabidopsis thaliana Hi-0	**4°C, pH 4.2**		**4°C, pH 6.4**		**4°C, pH 8.2**	4	**22°C, pH 4.2**		**22°C, pH 6.4**		**22°C, pH 8.2**		**37°C, pH 4.2**		**37°C, pH 6.4**		**37°C, pH 8.2**	
2-propenyl CN	**0.2353 ±**	0.0785	**0.4047 ±**	0.2263	**0.2097 ±**	0.0463	**0.1713 ±**	0.0961	**0.3074 ±**	0.0568	**0.1600 ±**	0.0186	**0.1952 ±**	0.0610	**0.1445 ±**	0.0464	**0.0848 ±**	0.0112
2-propenyl ITC	**0.9009 ±**	0.2021	**0.5600 ±**	0.1739	**0.8381 ±**	0.0522	**0.6106 ±**	0.1781	**0.6050 ±**	0.1447	**0.8405 ±**	0.2327	**0.5789 ±**	0.1284	**0.3421 ±**	0.0246	**0.6884 ±**	0.1199
1-cyano-2,3-epithiopropane	**0.0044 ±**	0.0008	**0.0031 ±**	0.0018	**0.0015 ±**	0.0007	**0.0056 ±**	0.0013	**0.0026 ±**	0.0010	**0.0010 ±**	0.0001	**0.0105 ±**	0.0031	**0.0010 ±**	0.0005	**0.0005 ±**	0.0002
3-butenyl ITC	**0.0036 ±**	0.0011	**0.0025 ±**	0.0013	**0.0034 ±**	0.0004	**0.0029 ±**	0.0016	**0.0028 ±**	0.0009	**0.0033 ±**	0.0014	**0.0037 ±**	0.0007	**0.0016 ±**	0.0001	**0.0034 ±**	0.0011
7-(methylthio)heptyl ITC	**0.0013 ±**	0.0002	**0.0037 ±**	0.0025	**0.0011 ±**	0.0002	**0.0014 ±**	0.0005	**0.0067 ±**	0.0010	**0.0010 ±**	0.0003	**0.0014 ±**	0.0003	**0.0063 ±**	0.0008	**0.0053 ±**	0.0004
8-(methylthio)octyl CN	**0.0007 ±**	0.0007	**0.0007 ±**	0.0008	**0.0007 ±**	0.0007	**0.0012 ±**	0.0006	**0.0011 ±**	0.0001	**0.0013 ±**	0.0001	**0.0013 ±**	0.0004	**0.0020 ±**	0.0003	**0.0014 ±**	0.0002
8-(methylthio)octyl ITC	**0.0123 ±**	0.0020	**0.0055 ±**	0.0023	**0.0082 ±**	0.0021	**0.0104 ±**	0.0039	**0.0042 ±**	0.0007	**0.0062 ±**	0.0024	**0.0112 ±**	0.0024	**0.0019 ±**	0.0004	**0.0033 ±**	0.0011
2-phenylethyl CN	**0.0010 ±**	0.0003	**0.0010 ±**	0.0003	**0.0008 ±**	0.0001	**0.0013 ±**	0.0001	**0.0010 ±**	0.0001	**0.0012 ±**	0.0003	**0.0012 ±**	0.0003	**0.0009 ±**	0.0007	**0.0011 ±**	0.0001
indole-3-acetonitrile	**0.0010 ±**	0.0001	**0.0022 ±**	0.0006	**0.0009 ±**	0.0009	**0.0008 ±**	0.0003	**0.0007 ±**	0.0006	**0.0003 ±**	0.0005	**0.0000 ±**	0.0000	**0.0005 ±**	0.0004	**0.0006 ±**	0.0006

Brassica oleracea BroccoCress®	**4°C, pH 3.8**		**4°C, pH 5.6**		**4°C, pH 8.2**		**22°C, pH 3.8**		**22°C, pH 5.6**		**22°C, pH 8.2**		**37°C, pH 3.8**		**37°C, pH 5.6**		**37°C, pH 8.2**	
4-(methylthio)butyl CN	**1.0418 ±**	0.1780	**0.9735 ±**	0.1604	**0.8010 ±**	0.2794	**0.9300 ±**	0.0330	**0.9218 ±**	0.1484	**0.7404 ±**	0.1278	**0.2935 ±**	0.0492	**1.0183 ±**	0.2372	**0.4334 ±**	0.0684
4-(methylthio)butyl ITC	**0.2701 ±**	0.2914	**0.1013 ±**	0.0382	**0.5326 ±**	0.0730	**0.3022 ±**	0.0991	**0.1301 ±**	0.0273	**0.4201 ±**	0.2004	**0.3974 ±**	0.0771	**0.0760 ±**	0.0499	**0.2453 ±**	0.0916
5-(methylthio)pentyl CN	**0.0008 ±**	0.0002	**0.0042 ±**	0.0009	**0.0032 ±**	0.0013	**0.0039 ±**	0.0002	**0.0042 ±**	0.0010	**0.0031 ±**	0.0008	**0.0016 ±**	0.0002	**0.0021 ±**	0.0009	**0.0016 ±**	0.0004
3-(methylsulphinyl)propyl CN	**0.0618 ±**	0.0496	**0.0710 ±**	0.1009	**0.0179 ±**	0.0047	**0.0280 ±**	0.0073	**0.0290 ±**	0.0118	**0.0191 ±**	0.0061	**0.0126 ±**	0.0052	**0.0286 ±**	0.0116	**0.0177 ±**	0.0129
3-(methylsulphinyl)propyl ITC	**0.0194 ±**	0.0140	**0.0125 ±**	0.0064	**0.0377 ±**	0.0066	**0.0285 ±**	0.0050	**0.0183 ±**	0.0041	**0.0519 ±**	0.0138	**0.0734 ±**	0.0104	**0.0367 ±**	0.0490	**0.1544 ±**	0.0962
4-(methylsulphinyl)butyl CN	**2.5013 ±**	0.7334	**1.9967 ±**	0.2595	**1.6946 ±**	0.2707	**2.1454 ±**	0.4056	**1.9604 ±**	0.3395	**1.0231 ±**	0.2523	**0.6481 ±**	0.0413	**1.2559 ±**	0.2341	**0.5151 ±**	0.0578
4-(methylsulphinyl)butyl ITC	**1.3149 ±**	1.5635	**0.4885 ±**	0.1468	**2.6549 ±**	0.1353	**1.6530 ±**	0.3352	**0.6452 ±**	0.1580	**2.0664 ±**	0.5858	**2.6445 ±**	0.2629	**0.8798 ±**	0.8123	**2.8103 ±**	0.9438
2-phenylethyl CN	**0.0287 ±**	0.0043	**0.0371 ±**	0.0043	**0.0230 ±**	0.0041	**0.0377 ±**	0.0034	**0.0456 ±**	0.0099	**0.0282 ±**	0.0059	**0.0283 ±**	0.0014	**0.0391 ±**	0.0088	**0.0222 ±**	0.0036
2-phenylethyl ITC	**0.0088 ±**	0.0096	**0.0062 ±**	0.0020	**0.0168 ±**	0.0047	**0.0105 ±**	0.0010	**0.0080 ±**	0.0040	**0.0153 ±**	0.0037	**0.0128 ±**	0.0017	**0.0040 ±**	0.0039	**0.0058 ±**	0.0042
indole-3-acetonitrile	**0.0511 ±**	0.0184	**0.0677 ±**	0.0179	**0.0153 ±**	0.0046	**0.0406 ±**	0.0045	**0.0542 ±**	0.0059	**0.0181 ±**	0.0064	**0.0187 ±**	0.0036	**0.0493 ±**	0.0190	**0.0159 ±**	0.0040
4-methoxyindole-3-acetonitrile	**0.0149 ±**	0.0045	**0.0448 ±**	0.0058	**0.0081 ±**	0.0031	**0.0065 ±**	0.0011	**0.0226 ±**	0.0033	**0.0047 ±**	0.0015	**0.0050 ±**	0.0016	**0.0153 ±**	0.0091	**0.0031 ±**	0.0012

Influence of temperature at pH 4.2 on GLS-degradation in Bur-0

pH 4.2	% of 2-propenyl glucosinolate breakdowns			% of 3-butenyl glucosinolate breakdowns		
T	2-propenyl CN	2-propenyl ITC	1-cyano-2,3-epithiopropane	3-butenyl CN	3-butenyl ITC	1-cyano-3,4-epithiobutane
4	2.99 ± 0.60 a	3.96 ± 0.81 a	93.05 ± 1.09 b	2.62 ± 1.32 a	9.28 ± 2.12 a	88.10 ± 2.36 b
22	4.20 ± 1.45 a	4.35 ± 0.39 a	91.45 ± 1.66 b	2.42 ± 0.95 a	8.74 ± 0.66 a	88.84 ± 1.15 b
37	14.42 ± 4.60 b	9.81 ± 1.47 b	75.77 ± 6.03 a	8.56 ± 2.48 b	16.21 ± 3.44 b	75.23 ± 5.89 a

Influence of dilution

µmol/g fresh weight

Brassica rapa 215	H_2O, 22°C, Dilutionfactor 2		H_2O, 22°C, Dilutionfactor 3		H_2O, 22°C, Dilutionfactor 9		H_2O, 22°C, Dilutionfactor 17		Saliva buffer, 37°C, Dilutionfactor 2		Saliva buffer, 37°C, Dilutionfactor 3		Saliva buffer, 37°C, Dilutionfactor 9		Inactivated Plant Material, 22°C, Dilutionfactor 2		Inactivated Plant Material, 22°C, Dilutionfactor 3		Inactivated Plant Material, 22°C, Dilutionfactor 9	
3-butenyl CN	**0.0100 ±**	0.0114	**0.0236 ±**	0.0216	**0.0117 ±**	0.0040	**0.0095 ±**	0.0019	**0.0037 ±**	0.0016	**0.0094 ±**	0.0051	**0.0037 ±**	0.0028	**0.0112 ±**	0.0064	**0.0146 ±**	0.0087	**0.0086 ±**	0.0028
3-butenyl ITC	**0.0081 ±**	0.0035	**0.0207 ±**	0.0239	**0.0490 ±**	0.0175	**0.0857 ±**	0.0134	**0.0052 ±**	0.0059	**0.0164 ±**	0.0056	**0.0480 ±**	0.0319	**0.0322 ±**	0.0191	**0.0229 ±**	0.0110	**0.0242 ±**	0.0083
1-cyano-3,4-epithiobutane	**0.2531 ±**	0.1539	**0.7289 ±**	0.6595	**0.2014 ±**	0.0593	**0.2096 ±**	0.0857	**0.2529 ±**	0.1555	**0.2625 ±**	0.0754	**0.0614 ±**	0.0482	**0.3092 ±**	0.0516	**0.1696 ±**	0.0369	**0.0760 ±**	0.0039
4-pentenyl ITC	**0.0032 ±**	0.0008	**0.0167 ±**	0.0205	**0.0198 ±**	0.0058	**0.0368 ±**	0.0101	**0.0065 ±**	0.0069	**0.0150 ±**	0.0053	**0.0149 ±**	0.0110	**0.0179 ±**	0.0093	**0.0139 ±**	0.0056	**0.0140 ±**	0.0037
1-cyano-4,5-epithiopentane	**0.0815 ±**	0.0505	**0.2048 ±**	0.2018	**0.0685 ±**	0.0273	**0.0708 ±**	0.0406	**0.1136 ±**	0.0879	**0.1236 ±**	0.0525	**0.0180 ±**	0.0156	**0.1168 ±**	0.0159	**0.0589 ±**	0.0145	**0.0177 ±**	0.0008
2-phenylethyl CN	**0.0085 ±**	0.0065	**0.0189 ±**	0.0154	**0.0047 ±**	0.0013	**0.0061 ±**	0.0010	**0.0068 ±**	0.0055	**0.0053 ±**	0.0021	**0.0025 ±**	0.0015	**0.0084 ±**	0.0021	**0.0044 ±**	0.0009	**0.0041 ±**	0.0005
2-phenylethyl ITC	**0.0010 ±**	0.0002	**0.0010 ±**	0.0002	**0.0015 ±**	0.0009	**0.0024 ±**	0.0002	**0.0017 ±**	0.0011	**0.0007 ±**	0.0004	**0.0012 ±**	0.0004	**0.0022 ±**	0.0019	**0.0021 ±**	0.0006	**0.0021 ±**	0.0004

Brassica rapa 374	H_2O, 22°C, Dilutionfactor 2		H_2O, 22°C, Dilutionfactor 3		H_2O, 22°C, Dilutionfactor 9		H_2O, 22°C, Dilutionfactor 17		Saliva buffer, 37°C, Dilutionfactor 2		Saliva buffer, 37°C, Dilutionfactor 3		Saliva buffer, 37°C, Dilutionfactor 9		Inactivated Plant Material, 22°C, Dilutionfactor 2		Inactivated Plant Material, 22°C, Dilutionfactor 9	
3-butenyl CN	**0.1675 ±**	0.0744	**0.0748 ±**	0.0256	**0.0580 ±**	0.0133	**0.0685 ±**	0.0345	**0.0844 ±**	0.0906	**0.0893 ±**	0.0130	**0.0953 ±**	0.0514	**0.1212 ±**	0.1023	**0.1417 ±**	0.0721
3-butenyl ITC	**0.3220 ±**	0.0855	**0.3049 ±**	0.0784	**0.5547 ±**	0.1364	**0.7084 ±**	0.1377	**0.1911 ±**	0.0773	**0.4784 ±**	0.2038	**0.8427 ±**	0.2416	**0.3084 ±**	0.0128	**0.5584 ±**	0.0520
1-cyano-3,4-epithiobutane	**2.1484 ±**	1.0656	**0.9903 ±**	0.6344	**0.9153 ±**	0.1746	**1.2581 ±**	0.9686	**1.1667 ±**	1.3534	**0.9389 ±**	0.2511	**0.8574 ±**	0.4033	**1.6961 ±**	1.0196	**0.9456 ±**	0.1443
4-pentenyl ITC	**0.1106 ±**	0.1359	**0.0830 ±**	0.0682	**0.0964 ±**	0.1000	**0.1534 ±**	0.0710	**0.0540 ±**	0.0776	**0.0286 ±**	0.0215	**0.0699 ±**	0.0262	**0.1162 ±**	0.0990	**0.0915 ±**	0.0234
1-cyano-4,5-epithiopentane	**0.4155 ±**	0.5233	**0.1654 ±**	0.1862	**0.0924 ±**	0.0966	**0.2649 ±**	0.2907	**0.3041 ±**	0.5016	**0.0337 ±**	0.0225	**0.0678 ±**	0.0469	**0.3931 ±**	0.5397	**0.0637 ±**	0.0242
2-phenylethyl CN	**0.0317 ±**	0.0176	**0.0107 ±**	0.0104	**0.0164 ±**	0.0119	**0.0274 ±**	0.0286	**0.0210 ±**	0.0250	**0.0159 ±**	0.0077	**0.0305 ±**	0.0173	**0.0218 ±**	0.0146	**0.0138 ±**	0.0051
2-phenylethyl ITC	**0.0169 ±**	0.0047	**0.0103 ±**	0.0052	**0.0184 ±**	0.0122	**0.0143 ±**	0.0036	**0.0129 ±**	0.0079	**0.0221 ±**	0.0156	**0.0369 ±**	0.0225	**0.0197 ±**	0.0069	**0.0159 ±**	0.0051

Brassica oleracea BroccoCress®	H_2O, 22°C, Dilutionfactor 2		H_2O, 22°C, Dilutionfactor 3		H_2O, 22°C, Dilutionfactor 9		H_2O, 22°C, Dilutionfactor 17		Saliva buffer, 37°C, Dilutionfactor 2		Saliva buffer, 37°C, Dilutionfactor 3		Saliva buffer, 37°C, Dilutionfactor 9		Inactivated Plant Material, 22°C, Dilutionfactor 2		Inactivated Plant Material, 22°C, Dilutionfactor 3		Inactivated Plant Material, 22°C, Dilutionfactor 9	
4-(methylthio)butyl CN	**1.0325 ±**	0.5561	**0.8792 ±**	0.0748	**0.8516 ±**	0.0864	**0.6837 ±**	0.0628	**0.6054 ±**	0.1479	**0.4173 ±**	0.1115	**0.5080 ±**	0.1149	**0.6604 ±**	0.0563	**0.5289 ±**	0.1111	**0.3903 ±**	0.0283
4-(methylthio)butyl ITC	**0.1069 ±**	0.0625	**0.0752 ±**	0.0201	**0.1387 ±**	0.0299	**0.2841 ±**	0.1167	**0.1303 ±**	0.0129	**0.1188 ±**	0.0457	**0.3812 ±**	0.0513	**0.1266 ±**	0.0021	**0.3200 ±**	0.0435	**0.4975 ±**	0.0170
5-(methylthio)pentyl CN	**0.0016 ±**	0.0011	**0.0014 ±**	0.0011	**0.0013 ±**	0.0001	**0.0015 ±**	0.0002	**0.0012 ±**	0.0009	**0.0005 ±**	0.0004	**0.0010 ±**	0.0001	**0.0007 ±**	0.0002	**0.0009 ±**	0.0002	**0.0011 ±**	0.0004
3-(methylsulfinyl)propyl CN	**0.0834 ±**	0.0865	**0.0194 ±**	0.0042	**0.0722 ±**	0.1132	**0.0095 ±**	0.0076	**0.0681 ±**	0.0930	**0.0145 ±**	0.0040	**0.0884 ±**	0.0721	**0.0066 ±**	0.0020	**0.1420 ±**	0.0719	**0.0648 ±**	0.0153
3-(methylsulfinyl)propyl ITC	**0.0149 ±**	0.0028	**0.0071 ±**	0.0024	**0.0149 ±**	0.0099	**0.0298 ±**	0.0208	**0.0843 ±**	0.0737	**0.0456 ±**	0.0147	**0.0416 ±**	0.0056	**0.0062 ±**	0.0003	**0.0368 ±**	0.0068	**0.0695 ±**	0.0117
4-(methylsulfinyl)butyl CN	**1.5311 ±**	0.5871	**1.0493 ±**	0.0879	**1.0230 ±**	0.1583	**0.8049 ±**	0.1577	**0.6940 ±**	0.0243	**0.4730 ±**	0.1841	**0.4046 ±**	0.0790	**0.5528 ±**	0.0129	**2.0638 ±**	0.1790	**1.2038 ±**	0.0830
4-(methylsulfinyl)butyl ITC	**0.3956 ±**	0.1355	**0.2429 ±**	0.0711	**0.4134 ±**	0.1314	**0.8561 ±**	0.4103	**1.3435 ±**	0.3060	**1.2220 ±**	0.2936	**1.6515 ±**	0.2846	**0.3671 ±**	0.0478	**0.9966 ±**	0.0735	**1.3868 ±**	0.1427
2-phenylethyl CN	**0.0310 ±**	0.0134	**0.0279 ±**	0.0063	**0.0305 ±**	0.0064	**0.0261 ±**	0.0033	**0.0235 ±**	0.0026	**0.0191 ±**	0.0029	**0.0223 ±**	0.0017	**0.0185 ±**	0.0012	**0.0225 ±**	0.0024	**0.0206 ±**	0.0010
2-phenylethyl ITC	**0.0047 ±**	0.0035	**0.0023 ±**	0.0003	**0.0047 ±**	0.0026	**0.0064 ±**	0.0028	**0.0055 ±**	0.0015	**0.0042 ±**	0.0009	**0.0073 ±**	0.0005	**0.0059 ±**	0.0004	**0.0117 ±**	0.0020	**0.0126 ±**	0.0012
indole-3-acetonitrile	**0.0393 ±**	0.0234	**0.0386 ±**	0.0066	**0.0530 ±**	0.0158	**0.0340 ±**	0.0029	**0.0143 ±**	0.0046	**0.0160 ±**	0.0060	**0.0146 ±**	0.0042	**0.0153 ±**	0.0016	**0.0145 ±**	0.0013	**0.0152 ±**	0.0014
4-methoxyindole-3-acetonitrile	**0.0312 ±**	0.0287	**0.0216 ±**	0.0076	**0.0100 ±**	0.0034	**0.0042 ±**	0.0013	**0.0029 ±**	0.0015	**0.0026 ±**	0.0012	**0.0025 ±**	0.0006	**0.0057 ±**	0.0003	**0.0052 ±**	0.0006	**0.0061 ±**	0.0006

Brassica oleracea white cabbage sprouts	H_2O, 22°C, Dilutionfactor 2		H_2O, 22°C, Dilutionfactor 3		H_2O, 22°C, Dilutionfactor 9		H_2O, 22°C, Dilutionfactor 17		Saliva buffer, 37°C, Dilutionfactor 2		Saliva buffer, 37°C, Dilutionfactor 3		Saliva buffer, 37°C, Dilutionfactor 9		Inactivated Plant Material, 22°C, Dilutionfactor 2		Inactivated Plant Material, 22°C, Dilutionfactor 3		Inactivated Plant Material, 22°C, Dilutionfactor 9	
2-propenyl CN	**0.2289 ±**	0.0790	**0.1277 ±**	0.0495	**0.0977 ±**	0.0399	**0.1169 ±**	0.0283	**0.2054 ±**	0.1136	**0.3157 ±**	0.0238	**0.1892 ±**	0.0467	**0.2322 ±**	0.0742	**0.1878 ±**	0.0707	**0.2267 ±**	0.0084
2-propenyl ITC	**0.1870 ±**	0.0870	**0.0876 ±**	0.0199	**0.1156 ±**	0.0457	**0.2967 ±**	0.0904	**0.1217 ±**	0.0704	**0.4041 ±**	0.0450	**0.6507 ±**	0.1453	**0.2641 ±**	0.0847	**0.2972 ±**	0.1047	**0.9247 ±**	0.0341
1-cyano-2,3-epithiopropane	**1.2233 ±**	0.2940	**0.9167 ±**	0.1769	**0.7394 ±**	0.2531	**0.8980 ±**	0.2288	**0.8401 ±**	0.1874	**0.7342 ±**	0.0532	**0.3595 ±**	0.0265	**0.8925 ±**	0.0240	**0.7615 ±**	0.3117	**0.2910 ±**	0.0264
2-hydroxy-3-butenyl CN	**0.0390 ±**	0.0080	**0.0222 ±**	0.0111	**0.0199 ±**	0.0095	**0.0293 ±**	0.0074	**0.0449 ±**	0.0271	**0.0639 ±**	0.0167	**0.0515 ±**	0.0049	**0.0525 ±**	0.0096	**0.1318 ±**	0.0115	**0.1351 ±**	0.0165
1-cyano-2-hydroxy-3,4-epithiobutane	**0.1347 ±**	0.0165	**0.0978 ±**	0.0391	**0.0783 ±**	0.0363	**0.1126 ±**	0.0355	**0.1325 ±**	0.0599	**0.1405 ±**	0.0407	**0.0777 ±**	0.0053	**0.1519 ±**	0.0122	**0.2472 ±**	0.0793	**0.1343 ±**	0.0192
5-vinyl-1,3-oxazolidine-2-thione	**0.0011 ±**	0.0006	**0.0005 ±**	0.0003	**0.0005 ±**	0.0003	**0.0016 ±**	0.0007	**0.0026 ±**	0.0019	**0.0137 ±**	0.0065	**0.0235 ±**	0.0077	**0.0064 ±**	0.0024	**0.0126 ±**	0.0044	**0.0292 ±**	0.0016
3-(methylthio)propyl CN	**0.0398 ±**	0.0176	**0.0280 ±**	0.0111	**0.0130 ±**	0.0081	**0.0232 ±**	0.0077	**0.0222 ±**	0.0057	**0.0259 ±**	0.0024	**0.0148 ±**	0.0057	**0.0312 ±**	0.0060	**0.0325 ±**	0.0109	**0.0335 ±**	0.0023
3-(methylthio)propyl ITC	**0.0117 ±**	0.0039	**0.0053 ±**	0.0027	**0.0043 ±**	0.0038	**0.0131 ±**	0.0059	**0.0029 ±**	0.0026	**0.0110 ±**	0.0029	**0.0174 ±**	0.0050	**0.0232 ±**	0.0070	**0.0332 ±**	0.0067	**0.0534 ±**	0.0016
1-cyano-3,4-epithiobutane	**0.0240 ±**	0.0115	**0.0158 ±**	0.0023	**0.0072 ±**	0.0033	**0.0115 ±**	0.0048	**0.0127 ±**	0.0049	**0.0106 ±**	0.0009	**0.0066 ±**	0.0008	**0.0178 ±**	0.0015	**0.0171 ±**	0.0062	**0.0094 ±**	0.0011
4-(methylthio)butyl CN	**0.0051 ±**	0.0027	**0.0030 ±**	0.0022	**0.0000 ±**	0.0000	**0.0020 ±**	0.0018	**0.0016 ±**	0.0014	**0.0024 ±**	0.0007	**0.0008 ±**	0.0013	**0.0036 ±**	0.0006	**0.0042 ±**	0.0012	**0.0048 ±**	0.0003
4-(methylthio)butyl ITC	**0.0015 ±**	0.0002	**0.0008 ±**	0.0003	**0.0003 ±**	0.0006	**0.0005 ±**	0.0009	**0.0009 ±**	0.0008	**0.0010 ±**	0.0009	**0.0015 ±**	0.0006	**0.0037 ±**	0.0011	**0.0066 ±**	0.0009	**0.0104 ±**	0.0006

Glucosinolate hydrolysis products in myrosinase inactivated plant material (µmol/g FW)

Brassica rapa 215	
3-butenyl ITC	0.0083 ± 0.0081
Brassica rapa 374	
3-butenyl ITC	0.0024 ± 0.0007
Brassica oleracea BroccoCress®	
4-(methylthio)butyl CN	0.0771 ± 0.0135
4-(methylthio)butyl ITC	0.0048 ± 0.0012
4-(methylsulfinyl)butyl CN	0.1747 ± 0.0150
2-phenylethyl CN	0.0095 ± 0.0004
indole-3-acetonitrile	0.0095 ± 0.0019
4-methoxyindole-3-acetonitrile	0.0054 ± 0.0013
Brassica oleracea white cabbage sprouts	
2-propenyl CN	0.0128 ± 0.0014
2-propenyl ITC	0.0014 ± 0.0024
2-hydroxy-3-butenyl CN	0.0436 ± 0.0012
3-(methylthio)propyl CN	0.0065 ± 0.0001
4-(methylthio)butyl CN	0.0022 ± 0.0001
4-(methylthio)butyl ITC	0.0004 ± 0.0007

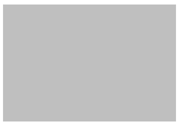

Supplement: Supplementary Information [file srep40807-s1.pdf]
